# Oral and gut microbiota dysbiosis with strengthened oral–gut connectivity in post-stroke cognitive impairment

**DOI:** 10.3389/fimmu.2026.1851811

**Published:** 2026-06-04

**Authors:** Xuetong Zhang, Fanping Chen, Jie Yang, Jiang Ma, Xiaoyan Li, Hui Wang, Qing Li, Yubin Zhao, Jianchao Xu

**Affiliations:** 1 Department of Rehabilitation Medicine, Shijiazhuang People’s Hospital, Shijiazhuang, China; 2 Department of Pharmacy, The Traditional Chinese Medicine Hospital of Shijiazhuang, Shijiazhuang, China; 3 Department of Acupuncture and Moxibustion, Hebei General Hospital, Shijiazhuang, China

**Keywords:** post-stroke cognitive impairment, oral microbiota, gut microbiota, oral–gut axis, gut oralization, oral enrichment score, machine learning, SHAP

## Abstract

**Background:**

Microbiome studies in post-stroke cognitive impairment (PSCI) have focused on the gut, while upstream oral dysbiosis and oral–gut axis signatures remain undercharacterized. We profiled paired oral and gut microbiota in PSCI to assess coordinated alterations and cross-site associations.

**Methods:**

This single-center cross-sectional study enrolled 133 post-stroke participants (64 PSCI). Paired tongue-coating and fecal samples underwent 16S rRNA gene sequencing. We compared α/β diversity, taxonomic composition, and predicted functional pathways at both sites, quantified within-individual oral–gut dissimilarity and the gut fraction of oral–gut shared microbiota, and adapted an oral enrichment score (OES) to index gut “oralization”. Differential microbiota, predicted pathways, and MMSE/MoCA scores were integrated into a multi-layer association framework. Machine-learning models were built using oral features, gut features, and combined oral–gut features, with SHAP for interpretability.

**Results:**

PSCI showed reduced oral richness (ACE/Chao1) and reduced gut diversity/evenness (Shannon/Simpson), with significant β-diversity differences at both sites. Oral commensals (*Leptotrichia*, *Neisseria*) were depleted, whereas opportunistic taxa (*Pseudomonas*, *Alloprevotella*, *Streptococcus*) were enriched. In the gut, SCFA-associated microbiota (*Coprococcus*, *Faecalibacterium*, *Ruminococcus*) decreased, while Gram-negative potential pathogens (*Enterobacter*, *Pseudomonas*, *Klebsiella*) increased. Tax4Fun2-based inference suggested predicted gut functional alterations involving lipopolysaccharide biosynthesis potential and tryptophan metabolism-related pathways. Oral–gut metrics supported stronger oral–gut association in PSCI, including lower paired dissimilarity, a higher shared-genera fraction, and elevated OES (median 0.0368 vs 0.0142), mainly driven by oral-dominant microbiota (*Streptococcus*, *Fusobacterium*, *Veillonella and Haemophilus*). Combined oral–gut features showed the most favorable exploratory test-set performance (XGBoost AUC 0.945; average precision 0.943).

**Conclusions:**

PSCI was associated with coordinated oral and gut dysbiosis, characterized by loss of commensals, enrichment of opportunistic or Gram-negative taxa, and a stronger gut oralization signal reflected by reduced oral–gut dissimilarity and elevated OES. Tax4Fun2 suggested predicted functional potential related to lipopolysaccharide biosynthesis and tryptophan metabolism, while combined oral–gut features showed favorable exploratory internal discrimination of PSCI. These findings require validation in larger longitudinal multi-omics cohorts.

## 1 Introduction

Post-stroke cognitive impairment (PSCI) is a common but frequently underestimated neuropsychological sequela. It persistently hinders functional recovery, quality of life, and social reintegration, while substantially increasing the burden on caregivers and healthcare systems (1). Although recent scientific statements emphasize managing risk factors, early identification, and standardized follow-up for PSCI (2, 3), its clinical onset and progression remain highly heterogeneous (4). Traditional risk factors and brain imaging structural indicators remain limited in predicting cognitive outcomes, and no reliable biomarkers are currently available (5). Consequently, the pathophysiology of PSCI likely extends beyond focal brain injury to involve dynamic changes and plasticity mechanisms at multiple systemic levels.

In this context, gut microbiota has emerged as a key peripheral regulatory system and a significant research focus for PSCI, owing to its intimate connections with immune-inflammatory responses, barrier homeostasis, and metabolite profiles. Clinical and mechanistic studies suggest an association between PSCI and gut microbial dysbiosis, where patients exhibit reduced community diversity, remodeling of specific bacterial taxa, and altered metabolites such as short-chain fatty acids that correlate with cognitive impairment severity and relevant clinical factors (6, 7). Reports on composite neuropsychiatric outcomes, such as co-occurring cognitive impairment and depression, further indicate that the gut microbiota may contribute to diverse neurological phenotypes after stroke (8). Critically, animal studies and interventions like fecal microbiota transplantation support a potential causal role, suggesting the gut microbiota influences post-stroke cognition via inflammation-modulating substances including lipopolysaccharide (LPS) and butyrate (9, 10). Collectively, this evidence suggests that gut dysbiosis may not only be an accompanying phenomenon of PSCI but could also participate in disease onset and progression via immune-metabolic pathways, thereby serving as a critical entry point for PSCI risk stratification and mechanistic intervention.

Research on the microbiome in PSCI has focused primarily on the gut microbiota, with systematic descriptions of the upstream oral microbiome remaining scarce. As the body’s second-largest and most diverse microbial habitat (11), the oral cavity can, through periodontal inflammation and pathogenic commensal expansion, disrupt distant organ homeostasis via swallowing, microbial migration, mucosal barrier breakdown, and inflammatory mediator spillover (12–14). The oral–gut axis concept (12, 15) is now widely used to explain such cross-site microbial interactions in multisystem diseases. This axis is closely linked to the development and progression of cardiometabolic disorders like hypertension, diabetes, and hyperlipidemia (16), as well as to various digestive conditions, including inflammatory bowel disease, chronic atrophic gastritis, and hepatocellular carcinoma (17–19). Notably, virulence factors from oral bacteria that translocate to the gut, and the immune-metabolic disturbances they provoke, may be connected to cognitive decline, suggesting a plausible oral-gut-brain pathway (20). Large-scale metagenomic analyses have recently introduced the oral enrichment score (OES) to gauge the overall enrichment of typical oral bacteria in the gut. Gut OES is consistently elevated across multiple disease states, indicating that “gut oralization” may reflect a conserved microbial dysbiosis signal (21). Concurrently, evidence linking the oral microbiota to stroke risk and prognosis continues to grow. Previous studies, however, have largely addressed stroke occurrence or general prognostic indicators (22, 23), leaving systematic investigations targeting PSCI as a specific neuropsychological outcome still needed. Even regarding oral health and post-stroke cognition, current evidence derives mainly from epidemiological associations between conditions like periodontitis and PSCI risk or severity, while the underlying microbiota-mediated mechanisms are unclear (24). Therefore, whether PSCI is characterized by coordinated dysbiosis across the oral and gut microbiota, and whether the oral–gut axis is associated with PSCI, warrants further investigation.

Based on this background, stroke patients were enrolled and stratified into post-stroke cognitive impairment (PSCI) and post-stroke normal cognition groups (PSNC) based on cognitive function. Paired oral and gut samples were collected for microbiome analysis to systematically characterize the differential features of the oral and gut microbiota. Furthermore, from the perspective of cross-ecological interactions, the interplay between oral and gut microbiota and its associative framework with functional pathways and cognitive scales was assessed.

## 2 Materials and methods

### 2.1 Study design and participants

This study was a single-center observational study with a cross-sectional sampling design. The participants were stroke patients hospitalized in the Department of Rehabilitation Medicine at Shijiazhuang People’s Hospital. The study protocol was approved by the Medical Ethics Committee of Shijiazhuang People’s Hospital (Approval No. 2024138), and written informed consent was obtained from all participants prior to enrollment.

Inclusion criteria: (1) Meeting the diagnostic criteria for stroke (25); (2) Age between 30 and 80 years; (3) Presence of focal neurological deficit signs, with the location of the responsible lesion confirmed by MRI/CT; (4) Time since stroke onset 3–6 months.

Exclusion criteria: (1) Coexisting cognitive impairment due to other causes such as Alzheimer’s disease, Parkinson’s disease, or frontotemporal dementia; (2) Pre-existing cognitive impairment prior to the stroke; (3) Inability to complete scale assessments due to impaired consciousness, aphasia, or psychiatric disorders; (4) Severe comorbid conditions with unstable vital signs; (5) Recent use of medications that may affect cognitive function (e.g., memantine hydrochloride, donepezil); (6) Coexisting oral diseases such as severe dental caries, severe periodontitis, or oral cancer; (7) Coexisting intestinal diseases such as ulcerative colitis, Crohn’s disease, or colon cancer; (8) Recent use of medications or agents that may affect the oral or gut microbiota, such as antibiotics, proton pump inhibitors, laxatives, glucocorticoids, probiotics, and mouthwash; (9) Inability to obtain tongue coating and fecal samples within 3 days after enrollment.

### 2.2 Clinical characteristics and sample collection

Following enrollment, experienced physicians from the Department of Rehabilitation Medicine collected demographic and stroke-related clinical data and cognitive function examinations. Participants were then categorized into PSCI or PSNC groups according to established diagnostic criteria (2). Two uniformly trained rehabilitation therapists, working independently and adhering to standardized protocols, administered all scale assessments. These assessments evaluated cognitive function using the Mini-Mental State Examination (MMSE) and Montreal Cognitive Assessment (MoCA) (26). Cognitive status was classified using predefined MMSE/MoCA-based criteria. For the MoCA, 1 point was added for participants with ≤12 years of formal education, and a corrected score <26 was considered abnormal, in accordance with the original MoCA scoring recommendations and official scoring instructions (27). For the MMSE, education-adjusted Chinese cutoffs were applied (≤17 for illiterate participants, ≤20 for primary school education, and ≤24 for junior high school education or above), consistent with prior studies in Chinese post-stroke populations (28).

Venous blood was drawn from fasting participants between 06:00 and 07:00 in the morning after enrollment and analyzed by the hospital laboratory. The measured parameters included red blood cell count (RBC), hemoglobin (HGB), lymphocyte count (LYM), white blood cell count (WBC), blood glucose (GLU), D-dimer, homocysteine (HCY), albumin (ALB), total cholesterol (TCHO), triglycerides (TG), high-density lipoprotein cholesterol (HDL-C), low-density lipoprotein cholesterol (LDL-C), apolipoprotein A1 (ApoA1), apolipoprotein B (ApoB), apolipoprotein E (ApoE), C-reactive protein (CRP), and erythrocyte sedimentation rate (ESR).

Tongue coating samples were collected under fasting conditions on the morning after enrollment. A trained operator used a sterile swab to gently scrape from the base to the tip of the tongue 30 times. The swab was placed into a sterile microcentrifuge tube containing 1 mL of phosphate-buffered saline (PBS) and vortexed to elute microorganisms. This process was repeated with two additional swabs to ensure sufficient sampling. The suspension was centrifuged for 15 min, after which the supernatant was discarded and the pellet retained. Samples were flash-frozen in liquid nitrogen and stored at −80°C.

Fecal samples were obtained using a sterile collection container, with participants instructed to avoid contamination from urine or toilet surfaces. Approximately 1–3 g of fecal material was collected from the interior of the stool using a sterile spoon and transferred to a sealed sterile microcentrifuge tube. All samples were flash-frozen in liquid nitrogen within two hours of collection and subsequently stored at −80°C.

### 2.3 16S rRNA sequencing and bioinformatics processing

Tongue-coating and fecal samples underwent 16S rRNA gene sequencing on an Illumina platform by Wuhan Metware Biotechnology Co., Ltd. (Wuhan, China). Microbial genomic DNA was extracted from the specimens using a CTAB-based method (29). The V3–V4 region of the bacterial 16S rRNA gene was amplified with sample-specific barcodes employing the universal primers 341F (5′-CCTACGGGNGGCWGCAG-3′) and 806R (5′-GGACTACHVGGGTWTCTAAT-3′). Following verification by agarose gel electrophoresis, amplicons were pooled at equimolar concentrations, purified, and prepared for library construction. These libraries were sequenced on an Illumina NovaSeq platform, generating 250-bp paired-end reads.

Raw reads were demultiplexed based on barcode sequences and trimmed to remove barcode and primer sequences. Paired-end reads were merged using FLASH v1.2.11 (30) and quality-filtered according to the QIIME v1.9.1 (31) workflow to generate clean tags. Chimeric sequences were removed to obtain effective tags for downstream analysis. Effective tags were clustered into operational taxonomic units (OTUs) at 97% sequence similarity using the UPARSE algorithm (32) implemented in USEARCH v7, and representative OTU sequences were taxonomically assigned using the Mothur method against the SILVA138.1 SSU rRNA database (https://www.arb-silva.de/), with a confidence threshold of 0.8–1.0. To mitigate bias from uneven sequencing depth, the OTU table was rarefied to an even depth of 57,460 reads per sample, corresponding to the minimum sequencing depth among all samples, before diversity and community-structure analyses. This procedure discarded 2,213,625 reads in total, with an average of 16,644 reads discarded per sample; no samples were excluded after rarefaction. A 97% OTU-based workflow was used because the present study focused primarily on genus-level microbial composition, diversity, oral–gut overlap, OES calculation, and predicted functional profiles rather than strain- or sequence-variant-level inference.

### 2.4 Microbiome statistical analyses

Bioinformatic and statistical analyses of the OTU table and related data were performed in R (version 4.4.2). Taxonomic annotation was completed using the *microeco* package, after which sequences identified as potential contaminants and entries with uncertain taxonomic assignments were removed during data quality control. Relative abundances were calculated at different taxonomic levels, including phylum and genus. Community diversity and richness were assessed by calculating α-diversity indices (Shannon, Simpson, ACE, and Chao1) with the *microeco* package. The Bray–Curtis distance between samples was computed using the *vegan* package, and principal coordinate analysis (PCoA) was performed on this distance matrix to visualize differences in community structure. Permutational multivariate analysis of variance (PERMANOVA) was used to test for overall community structure differences between groups. Stacked bar charts of relative abundances at the phylum and genus levels were generated with *ggplot2*. Differences in the relative abundances of bacterial genera between two groups were assessed using the Wilcoxon rank-sum test. To control for false positives from multiple comparisons, *P* values were adjusted for multiple comparisons using the Benjamini–Hochberg FDR method and are reported as q values. The log2 fold change (log2FC) of the mean difference between groups was calculated, and a 95% confidence interval was derived via bootstrap resampling (n = 500). Group-specific taxonomic units were compared using set analysis with the *VennDiagram* package to generate Venn diagrams. Spearman correlation coefficients among scale scores, laboratory indicators, predicted functional pathway abundances, and genus-level abundances were calculated using the Hmisc package. *P* values were adjusted for multiple comparisons using the Benjamini–Hochberg false discovery rate (FDR) method and are reported as q values. The resulting correlation matrix was subjected to hierarchical clustering, and a correlation heatmap was plotted with *pheatmap* to visualize association patterns. The full-cohort differential abundance analysis was used solely for descriptive microbiome characterization and was not involved in the machine-learning feature-selection process.

### 2.5 Functional prediction and analysis

Functional profiles of the microbiota were inferred using the Tax4Fun2 algorithm (33) implemented in the *microeco* package. The NCBI BLAST+ suite served as the sequence alignment engine, and the Ref99NR reference database was used. Representative OTU sequences were aligned via BLAST to obtain KEGG Orthology (KO) annotations, which were aggregated to produce predicted abundance matrices for both KOs and KEGG pathways. These pathway results were summarized according to the KEGG hierarchy (Levels 1–3) using the built-in Tax4Fun2–KEGG mapping table. The predicted functional abundances were normalized to relative abundances per sample, yielding final relative abundance matrices for each hierarchical level. These Tax4Fun2-derived outputs were interpreted as predicted microbial functional potential rather than direct measurements of metabolic flux, gene expression, or *in vivo* metabolite levels.

Stacked bar charts illustrating the KEGG Level 2 functional composition of the PSCI and PSNC groups were generated from the Tax4Fun2-predicted relative abundances to visualize overall functional differences. Functional alpha diversity was assessed by calculating the Shannon and Simpson indices with the *vegan* package. Bray–Curtis distances based on functional profiles were also computed, and PCoA was applied to these distances to examine community structure differences. PERMANOVA was used to test for overall group differences. Differential analysis of Tax4Fun2-inferred predicted KEGG Level 3 pathways was performed using the Wilcoxon rank-sum test, with *P* values adjusted for multiple comparisons using the Benjamini–Hochberg false discovery rate (FDR) method and reported as q values. Effect sizes were expressed as log2FC based on group medians. Significantly differential pathways were visualized using *volcano* and *raincloud* plots. Finally, Spearman correlations between clinical scales/laboratory indicators and differential predicted KEGG pathway abundances were assessed and visualized using heatmaps and scatter plots; group-stratified analyses were further performed within the PSCI and PSNC groups, with *P* values adjusted using the Benjamini–Hochberg FDR method and reported as q values.

### 2.6 Oral–gut axis analyses

All analyses of the oral–gut axis were performed on paired oral and gut samples from the same subjects, using relative abundance data at the genus level. The compositional overlap of the oral and gut microbiota between the PSCI and PSNC groups was characterized by tallying taxonomic units detected in the oral cavity and gut at the phylum and genus levels, which were visualized with the *VennDiagram* package. To evaluate whether Venn-based shared-taxa comparisons were affected by post-rarefaction coverage differences, Good’s coverage was summarized by group and sample type and compared between PSCI and PSNC using the Wilcoxon rank-sum test. For each individual, the Bray–Curtis and Jaccard distances between their oral and gut samples were calculated. Distance matrices were computed using the *vegan* package, and PCoA was performed to illustrate trends in overall community structure separation. Permutation testing with PERMANOVA assessed differences in overall community composition between groups, while the Wilcoxon rank-sum test compared the within-individual paired Bray–Curtis and Jaccard distances between the PSCI and PSNC groups. Because Bray–Curtis dissimilarity is abundance-weighted and may be influenced by community evenness, diversity-adjusted linear regression models were further constructed. Jaccard distance was also analyzed as a complementary presence/absence-based distance metric. Genera shared between the oral and gut microbiota were identified. For each subject, the relative abundances of these shared genera in the gut sample were summed to yield the total abundance of oral–gut shared genera in the gut, and group differences in this metric were evaluated with the Wilcoxon rank-sum test.

The Oral Enrichment Score (OES) (21) quantified the overall enrichment of typical oral bacteria in each gut sample. Typical oral genera were defined as those with a relative abundance exceeding 1% and a prevalence over 30% in oral samples. To assess whether the OES result was dependent on the predefined threshold, we performed sensitivity analyses using alternative oral mean-abundance cutoffs of 0.5%, 1.0%, and 2.0% and oral prevalence cutoffs of 20%, 30%, and 40%. For each threshold combination, OES was recalculated and compared between PSCI and PSNC using the Wilcoxon rank-sum test, with Benjamini–Hochberg correction applied across threshold combinations. The OES for a gut sample was calculated as the sum of the relative abundances of these typical oral genera detected within it. The relative abundance of each typical oral genus in the gut represented its contribution to the OES. The average contribution and contribution proportion of each genus were calculated per group, and the Wilcoxon rank-sum test with Benjamini–Hochberg false discovery rate correction identified key genera driving OES differences between groups. Spearman correlation analysis assessed associations between the oral and gut microbiota and between OES-associated genera and differentially abundant functional pathways. To explore potential cascading relationships, data on oral microbiota, gut microbiota, functional pathways, and cognitive scale scores were integrated to construct a four-layer association chain. A Sankey diagram was used to visualize potential sequential links along the oral–gut–metabolic pathway–cognition axis. Fisher’s exact test was applied to assess the overrepresentation of significant associations between adjacent layers, and flow width was scaled according to −log10-transformed Fisher’s exact *P* values, with wider flows indicating stronger statistical enrichment.

### 2.7 Machine learning and interpretability

To avoid information leakage, machine-learning analyses were repeated using the original genus-level abundance tables rather than the full-cohort differential taxa. Participants were first randomly divided into a train set and a test set in a 7:3 ratio using stratified sampling according to PSCI status. The test set was not used for feature filtering, feature selection, hyperparameter tuning, model comparison, threshold determination, or any other model-development step, and was reserved only for final performance evaluation. We employed three distinct feature sets: oral-only, gut-only, and a combined oral–gut set. For the combined scheme, genus-level features from the oral and gut compartments of each subject were concatenated, with prefixes (e.g., oral_ and gut_) appended to preserve their origin and prevent naming conflicts.

Within each feature-input scheme, candidate microbial features were selected exclusively in the train set. First, robust feature selection was conducted using the Boruta algorithm (34) with parameters set to maxRuns = 500 and ntree = 1500 for the *Random Forest*, which retained only features confirmed as significant against shadow attributes. Subsequently, LASSO logistic regression (35) via *glmnet* was applied for further dimensionality reduction and to improve model generalizability, employing 10-fold cross-validation with binomial deviance as the loss function. Features with non-zero coefficients at the selected penalty parameter were retained for model construction. The test set was not used during Boruta selection or LASSO regression.

Six supervised learning models were then built and compared using these selected features. Model training, hyperparameter tuning, and exploratory algorithm comparison were conducted exclusively within the train set using repeated 5-fold cross-validation repeated 20 times. The models included: Logistic Regression (LR) (36) using *glmnet* to optimize alpha and lambda. Random Forest (RF) (37) implemented with ranger using the Gini index, 500 trees, and tuning for *mtry* and *min.node.size*. Support Vector Machine (SVM) (38) with a radial basis kernel. Neural Network (NNET) (39) using *nnet* with tuning for hidden layer size and weight decay. XGBoost (40), where the optimal boosting rounds were determined by cross-validation with early stopping and scale_pos_weight adjusted for class distribution. LightGBM, trained with early stopping and parameter grid search. The test set was reserved only for final evaluation.

Model performance was assessed through internal validation on the train set and validation on the independent test set. Receiver Operating Characteristic (ROC) curves were generated and the area under the curve (AUC) was calculated using the *pROC* package. To complement the ROC analysis, Precision–Recall (PR) curves were plotted on the test set, and the average precision (AP) was computed using the *PRROC* package, providing a focused evaluation of positive class (PSCI) identification. The 95% confidence intervals for AUC and AP were estimated using stratified bootstrap resampling with 2,000 resamples. Threshold-dependent clinical metrics included sensitivity, specificity, accuracy, balanced accuracy, positive predictive value, negative predictive value, and F1 score. Probabilistic performance and calibration were evaluated using the Brier score and LogLoss.

For model interpretation, Shapley Additive Explanations (SHAP) (41) were employed to quantify feature contributions, with global importance summarized by mean(|SHAP|) and visualized using bar plots, while feature-level directional effects across individual samples were illustrated using SHAP beeswarm plots. Because multiple algorithms were compared without a prespecified clinical prediction model, the machine-learning analysis was considered exploratory and was intended to evaluate internal discriminatory potential rather than establish a clinically validated diagnostic classifier.

### 2.8 Statistical analysis

All statistical analyses and visualizations were conducted using R (v4.4.2). Continuous variables are expressed as mean ± standard deviation or median (interquartile range, IQR), while categorical variables are reported as frequency (percentage). Continuous variables were compared between groups using either the independent samples t-test for normally distributed data or the Wilcoxon rank-sum test for non-normally distributed data. Categorical variables were analyzed with the chi-square test or Fisher’s exact test when expected frequencies were low. All tests were two-sided. For single comparisons, statistical significance was defined as *P* < 0.05. For analyses involving multiple comparisons, FDR-adjusted q < 0.05 was considered statistically significant.

## 3 Results

### 3.1 Clinical and demographic characteristics of the study cohort

**Figure 1:**
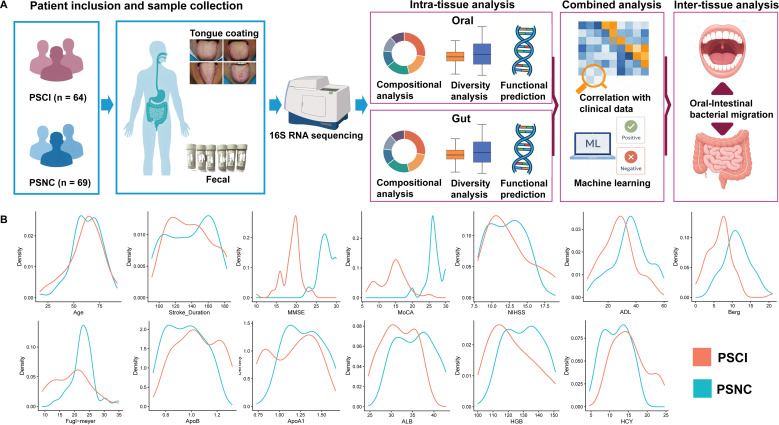
Overview of the study design and patient characteristics. **(A)** Flowchart illustrating the inclusion of post-stroke cognitive impairment (PSCI) patients (n = 64) and post-stroke normal cognition (PSNC) patients (n = 69). Tongue coating and fecal samples were collected for analysis. Samples underwent 16S rRNA sequencing for microbial community profiling. **(B)** Density plots comparing the distribution of clinical and demographic parameters between the PSCI and PSNC groups. MMSE, mini-mental state examination; MoCA, Montreal cognitive assessment; ApoA1, apolipoprotein A1; ApoB, apolipoprotein B; HCY, homocysteine; HGB, hemoglobin.

Between January 21, 2024, and August 21, 2025, 133 eligible stroke patients were enrolled, including 64 PSCI and 69 PSNC (**Figure 1A**). **Table 1** and **Figure 1** summarize the demographic and clinical characteristics of these patients. Baseline demographic and comorbidity indicators did not differ significantly between the two groups. The mean age was 61.52 ± 16.21 years for the PSCI group and 62.77 ± 13.89 years for the PSNC group. Females accounted for 64.06% and 75.36% of each group, respectively. At enrollment, stroke duration was 138.70 ± 25.80 days in the PSCI group and 136.77 ± 26.98 days in the PSNC group, a difference that was not statistically significant. No significant difference was observed in NIHSS scores between the PSCI group (12.50 ± 3.14) and the PSNC group (12.09 ± 2.50), suggesting a comparable degree of neurological impairment between the two groups at baseline.

**Table 1:** Characteristics of the enrolled patients.

Characteristics	PSCI (N = 64)	PSNC (N = 69)	*P* value
Demographic features			
Age, year	61.52 ± 16.21	62.77 ± 13.89	0.634
Female sex, n (%)	41 (64.06%)	52 (75.36%)	0.156
Education, low/middle/high, n	28/31/5	29/30/10	0.469
Weight, kg, (mean ± std)	70.08 ± 11.37	70.34 ± 11.99	0.778
Height, cm, (mean ± std)	165.20 ± 8.46	165.51 ± 9.01	0.834
BMI (mean ± std)	25.45 ± 1.56	25.56 ± 1.66	0.710
Smoking, n (%)	37 (57.81%)	39 (56.52%)	0.881
Drinking, n (%)	22 (34.38%)	26 (37.68%)	0.692
Stroke-related characteristics			
Stroke type, ischemic, n (%)	57 (78.13%)	47 (68.12%)	0.194
Stroke duration, days, (mean ± std)	138.70 ± 25.80	136.77 ± 26.98	0.695
WMH, n (%)	12 (18.75%)	15 (21.74%)	0.669
History of stroke, n (%)	12 (18.75%)	8 (11.59%)	0.259
NIHSS (mean ± std)	12.50 ± 3.14	12.09 ± 2.50	0.705
Comorbidities			
Hypertension, n (%)	51 (79.69%)	56 (81.16%)	0.831
Diabetes, n (%)	23 (35.93%)	19 (27.54%)	0.298
Hyperlipemia, n (%)	22 (34.38%)	21 (30.43%)	0.627
Cardiovascular disease, n (%)	15 (23.44%)	14 (20.29%)	0.660
Atrial fibrillation, n (%)	6 (9.38%)	2 (2.90%)	0.153
Kidney disease, n (%)	1 (1.56%)	0 (0.00%)	0.481
Liver disease, n (%)	6 (9.38%)	2 (2.90%)	0.153
Cognitive features			
MMSE (mean ± std)	18.94 ± 2.36	27.43 ± 1.88	<0.001^*^
MoCA (mean ± std)	14.19 ± 4.17	26.38 ± 2.38	<0.001^*^

BMI, Body Mass Index; NIHSS, National Institutes of Health Stroke Scale; MMSE, Mini-Mental State Examination; MoCA, Montreal Cognitive Assessment; WMH, White Matter Hyperintensities.

Bold values indicate P < 0.05. * P < 0.05.

Cognitive assessments using the MMSE and MoCA revealed significant differences between the groups (**Table 1**; **Figure 1B**). Laboratory results are presented in **Table 2** and **Figure 1**. Relative to the PSNC group, the PSCI group had significantly lower HGB, ALB and ApoA1 levels, while HCY and ApoB levels were significantly higher (**Table 2**; **Figure 1B**).

**Table 2:** Laboratory findings of the enrolled patients.

Laboratory data	PSCI (N = 64)	PSNC (N = 69)	*P* value
RBC,10^12^/L	4.30 ± 0.47	4.40 ± 0.79	0.532
HGB, g/L	122.59 ± 14.21	130.00 ± 11.89	0.002^*^
LYM, 10^9^/L	1.87 ± 0.74	1.85 ± 0.67	0.789
WBC, 10^9^/L	8.38 ± 2.87	8.21 ± 2.95	0.770
GLU, mmol/L	6.99 ± 1.77	6.53 ± 1.53	0.103
D-dimer, μg/L	500.00(323.75)	399.00(396.00)	0.170
HCY, μmol/L	14.66(6.68)	12.05(5.88)	<0.001^*^
ALB, g/L	31.70 ± 3.86	35.48 ± 4.29	<0.001^*^
TCHO, mmol/L	3.58 ± 1.45	3.54 ± 1.45	0.841
TG, mmol/L	1.84 ± 0.69	1.79 ± 0.66	0.741
HDL-C, mmol/L	1.07 ± 0.20	1.05 ± 0.17	0.315
LDL-C, mmol/L	2.48 ± 0.72	2.31 ± 0.68	0.198
ApoA1, g/L	1.16 ± 0.27	1.29 ± 0.22	0.009^*^
ApoB, g/L	1.05 ± 0.16	0.95 ± 0.14	0.001^*^
ApoE, mg/L	46.37 ± 11.77	47.62 ± 12.92	0.564
CRP, mg/L	8.76 ± 4.49	8.43 ± 3.84	0.719
ESR, mm/h	17.76 ± 8.77	17.69 ± 8.42	0.946

RBC, Red Blood Cells; HGB, Hemoglobin; LYM, Lymphocyte count; WBC, White Blood Cells; GLU, Glucose; HCY, Homocysteine; ALB, Albumin; TCHO, Total Cholesterol; TG, Triglycerides; HDL-C, High-Density Lipoprotein Cholesterol; LDL-C, Low-Density Lipoprotein Cholesterol; ApoA1, Apolipoprotein A1; ApoB, Apolipoprotein B; ApoE, Apolipoprotein E; CRP, C-Reactive Protein; ESR, Erythrocyte Sedimentation Rate. ^*^P < 0.05.

Bold values indicate P < 0.05.

### 3.2 Alterations in oral and gut microbiota diversity in PSCI

**Figure 2:**
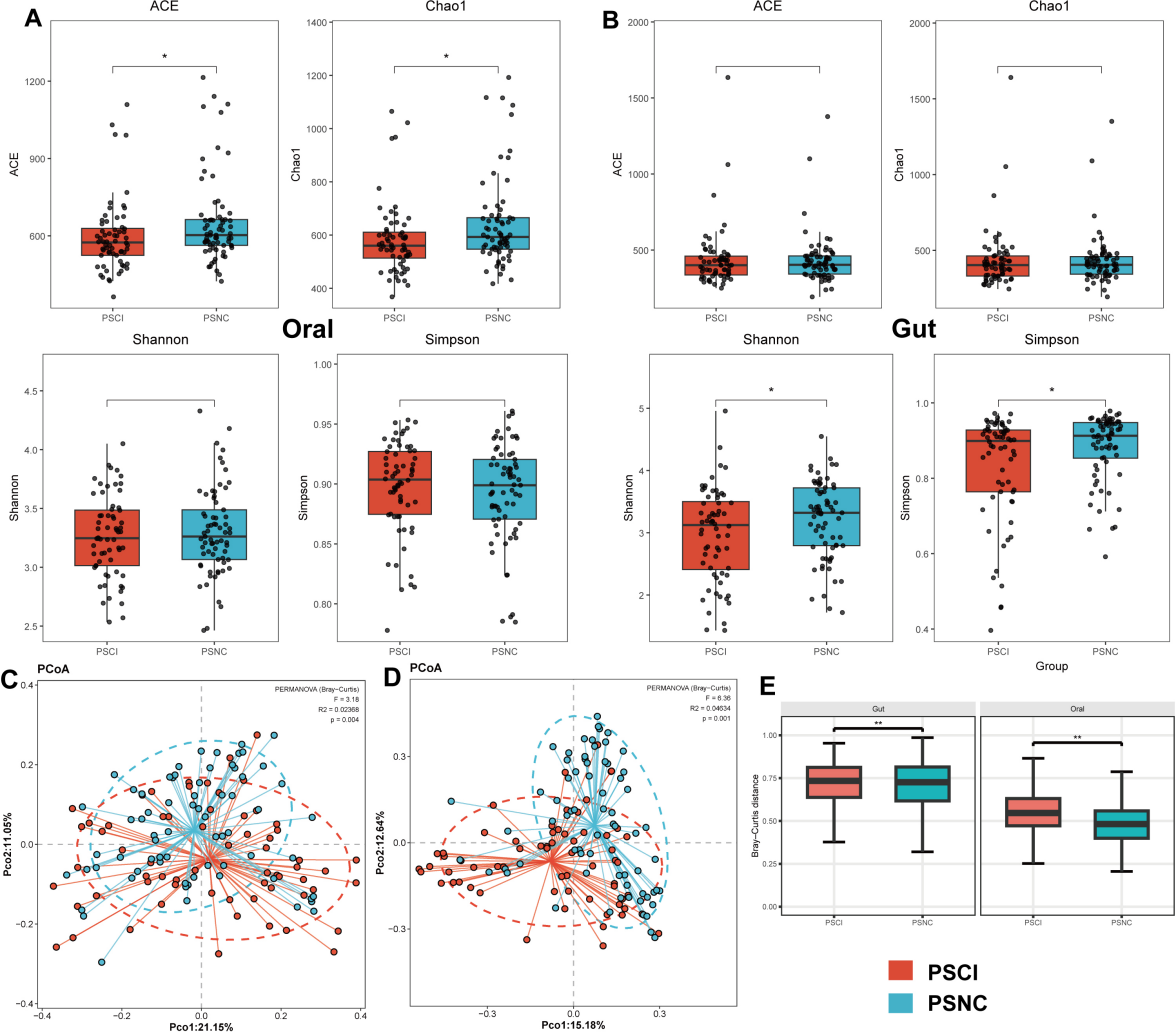
Microbial diversity analysis of oral and gut microbiota in PSCI and PSNC patients. **(A, B)** Alpha diversity indices: Boxplots displaying the richness (ACE and Chao1 indices) and diversity (Shannon and Simpson indices) of oral **(A)** and gut **(B)** microbiota in PSCI (red) and PSNC (blue) patients. Statistically significant differences are indicated by asterisks (*P* < 0.05). **(C, D)** PCoA plots based on Bray-Curtis distance showing the clustering of oral **(C)** and gut **(D)** microbiota between PSCI and PSNC groups. PERMANOVA test results are shown, indicating significant differences in microbial composition between groups (*P* < 0.05). **(E)** Beta diversity comparison: Boxplot comparing the Bray-Curtis distance between PSCI and PSNC groups for both oral and gut microbiota, showing significant differences in microbial community structure (*P* < 0.01).

Alpha diversity of the oral and gut microbiota was altered in the PSCI group. The ACE and Chao1 indices for oral microbiota were significantly lower in the PSCI group than in the PSNC group (both *P* < 0.05), whereas the Shannon and Simpson indices showed no significant difference (**Figure 2A**). For the gut microbiota, the Shannon and Simpson indices were significantly lower in the PSCI group (both *P* < 0.05), but the ACE and Chao1 indices did not differ significantly (**Figure 2B**). These results indicate that oral microbiota in PSCI patients exhibit reduced species richness but maintain community evenness and overall diversity, while gut microbiota shows no change in richness but decreased evenness and diversity.

PCoA based on Bray–Curtis distance revealed distinct community structures between the PSCI and PSNC groups for both oral and gut microbiota (**Figures 2C, D**). PERMANOVA confirmed statistically significant compositional differences between the groups (oral: F = 3.18, R² = 0.02368, *P* = 0.004; gut: F = 6.36, R² = 0.04634, *P* = 0.001). Furthermore, inter-group comparisons of Bray–Curtis distances were significantly different for both oral and gut microbiota (both *P* < 0.01) (**Figure 2E**).

### 3.3 Taxonomic shifts in the oral and gut microbiota associated with PSCI

Significant differences in the composition of the oral and gut microbiota were observed between the PSCI and PSNC groups at both the phylum and genus levels. At the phylum level, one oral phylum was unique to the PSCI group, while five were specific to the PSNC group. Within the gut microbiota, two group-specific phyla were identified in each of the PSCI and PSNC groups. Analysis at the genus level showed that the PSCI and PSNC groups contained 39 and 101 group-specific oral genera, respectively. For the gut microbiota, 73 group-specific genera were found in the PSCI group, compared to 39 in the PSNC group (**Supplementary Figure 1A**).

**Figure 3:**
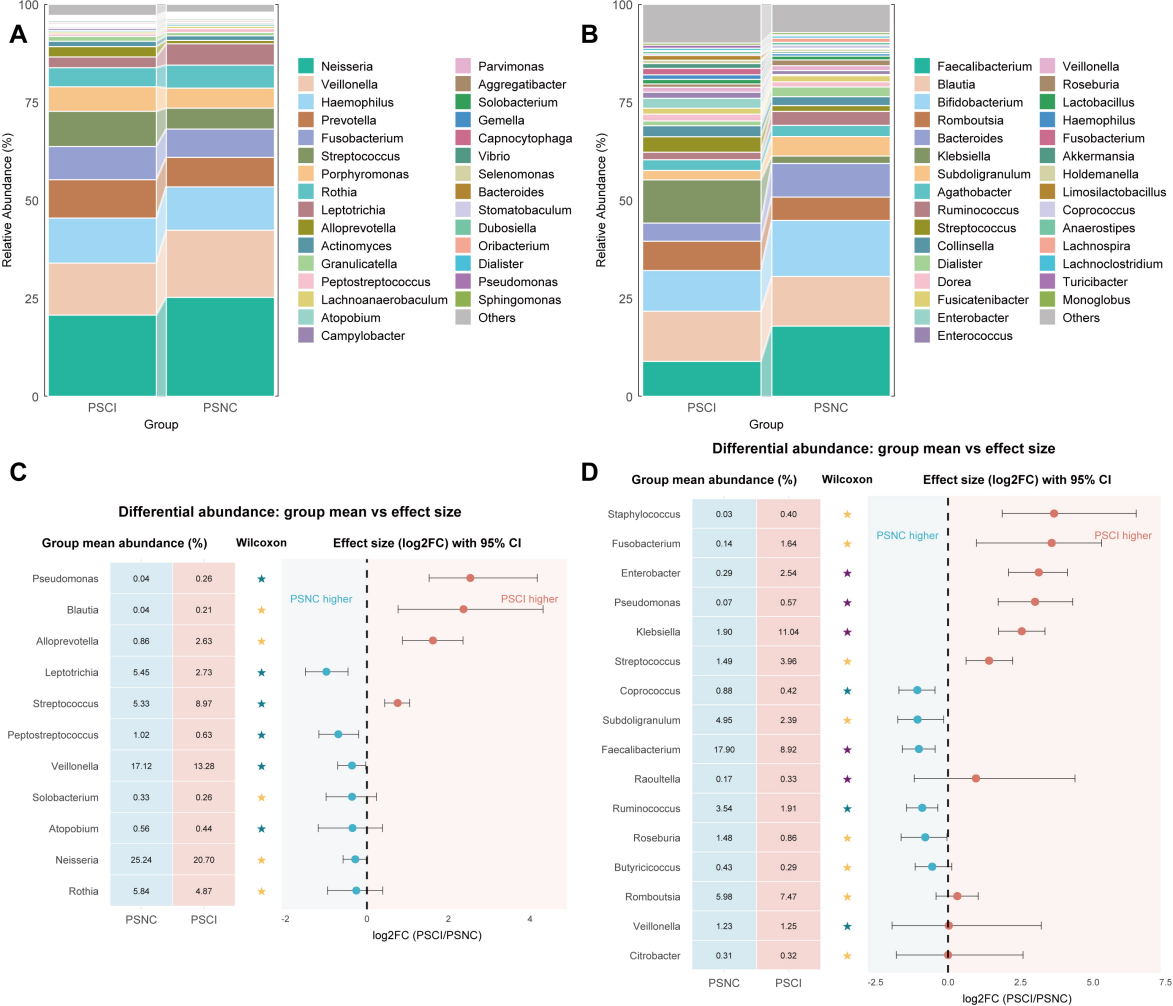
Microbial abundance differences between the PSCI and PSNC groups in oral and gut microbiota. **(A, B)** Relative abundance stacked bar plots: The stacked bar plots represent the relative abundance of bacterial genera in the oral **(A)** and gut **(B)** microbiota of PSCI and PSNC patients. Each genus is color-coded, and the height of each bar reflects the percentage of abundance for that genus in each group. **(C, D)** Differential abundance comparison of representative genera in the oral microbiota **(C)** and gut microbiota **(D)**. The left panels show the mean relative abundance (%) in each group. The middle column indicates Wilcoxon test (BH) significance using colored asterisks (yellow, q < 0.05; blue, q < 0.01; purple, q < 0.001). The right panels show effect sizes expressed as log2 fold change [log2FC (PSCI/PSNC)] with 95% confidence intervals. Values greater than 0 indicate enrichment in PSCI, whereas values less than 0 indicate enrichment in PSNC. The dashed vertical line represents log2FC = 0.

The oral microbiota at the phylum level was dominated by *Bacteroidota*, *Proteobacteria*, *Firmicutes*, *Fusobacteriota*, *Actinobacteria*, and *Gracilibacteria*, while the gut microbiota was primarily composed of *Firmicutes*, *Proteobacteria*, *Actinobacteria*, *Bacteroidota*, *Actinobacteriota*, and *Verrucomicrobiota* (**Supplementary Figures 1B, C**). At the genus level, the predominant oral genera included *Neisseria*, *Veillonella*, *Haemophilus*, *Prevotella*, *Fusobacterium*, *Streptococcus*, *Porphyromonas*, *Rothia*, *Leptotrichia*, and *Alloprevotella* (**Figure 3A**; **Supplementary Figure 1B**). The dominant gut genera were chiefly *Faecalibacterium*, *Blautia*, *Bifidobacterium*, *Romboutsia*, *Bacteroides*, *Klebsiella*, *Subdoligranulum*, *Agathobacter*, *Ruminococcus*, and *Streptococcus* (**Figure 3B**; **Supplementary Figure 1C**).

To identify intergroup differences, we further screened microbial taxa with a relative abundance of at least 0.1% at the genus level. In the oral microbiota, the PSCI group exhibited significantly higher relative abundances of *Pseudomonas*, *Blautia*, *Alloprevotella*, and *Streptococcus* (q < 0.05). Conversely, significantly greater relative abundances of *Leptotrichia*, *Peptostreptococcus*, *Veillonella*, *Solobacterium*, *Atopobium*, *Neisseria* and *Rothia* were observed in the PSNC group (q < 0.05) (**Figure 3C**). Within the gut microbiota, the relative abundances of *Staphylococcus*, *Fusobacterium*, *Enterobacter*, *Pseudomonas*, *Klebsiella*, *Streptococcus*, *Raoultella, Romboutsia*, *Veillonella*, and *Citrobacter* were significantly elevated in the PSCI group (q < 0.05). In contrast, the PSNC group showed significantly higher relative abundances of *Coprococcus*, *Subdoligranulum*, *Faecalibacterium*, *Ruminococcus*, *Roseburia*, and *Butyricicoccus* (q < 0.05) (**Figure 3D**).

### 3.4 Associations of the oral and gut microbiota with clinical parameters in PSCI

**Figure 4:**
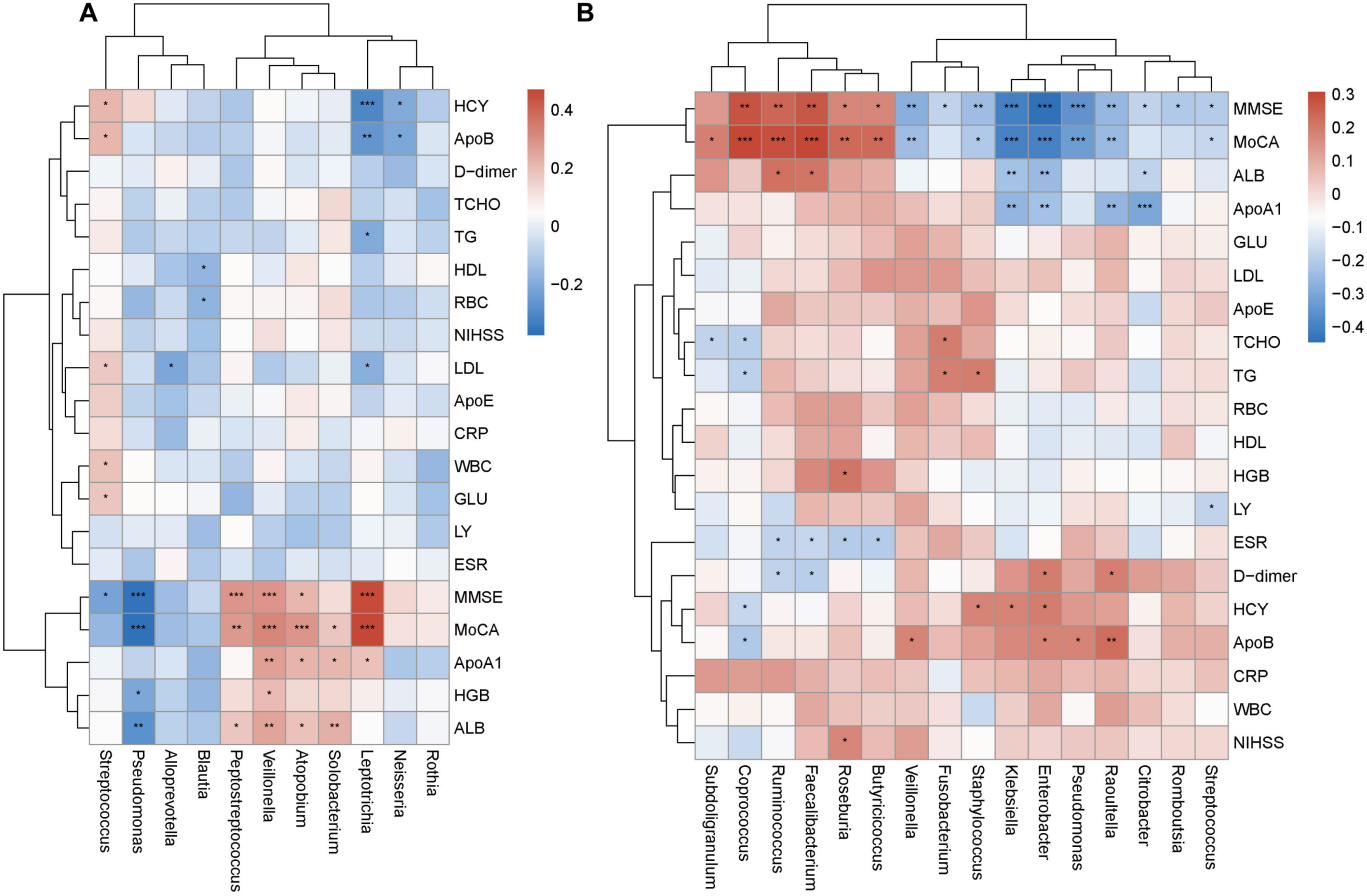
Associations between differential oral/gut genera and clinical characteristics in post-stroke patients. **(A)** Spearman’s rank correlation heatmap showing relationships between the relative abundances of selected oral microbiota and clinical/laboratory variables. **(B)** Spearman’s rank correlation heatmap showing relationships between the relative abundances of selected gut microbiota and clinical/laboratory variables. Color intensity indicates the correlation coefficient, with red denoting positive correlations and blue denoting negative correlations. Hierarchical clustering was applied to both microbiota and clinical variables to illustrate correlation-based grouping patterns. Asterisks indicate statistical significance (*q < 0.05, **q < 0.01, ***q < 0.001). *P* values were adjusted using the Benjamini–Hochberg FDR method (q values). MMSE, mini-mental state examination; MoCA, Montreal cognitive assessment; NIHSS, National Institutes of Health Stroke Scale; ALB, albumin; HGB, hemoglobin; HCY, homocysteine; CRP, C-reactive protein; ESR, erythrocyte sedimentation rate; GLU, glucose; RBC, red blood cell count; WBC, white blood cell count; TCHO, total cholesterol; TG, triglycerides; HDL, high-density lipoprotein; LDL, low-density lipoprotein; ApoA1/ApoB/ApoE, apolipoprotein A1/B/E.

Spearman correlation analysis assessed the associations between oral-gut microbiota and cognitive function scores or laboratory indicators, revealing several significant correlations (q < 0.05). For the oral microbiota, MMSE and MoCA scores correlated negatively with the relative abundances of *Streptococcus* and *Pseudomonas*, but positively with those of *Leptotrichia*, *Peptostreptococcus*, *Veillonella* and *Atopobium* (**Figure 4A**). HCY and ApoB levels correlated negatively with the relative abundances of *Leptotrichia* and *Neisseria*, and positively with that of *Streptococcus*. LDL, WBC, GLU, HCY and ApoB also showed a positive correlation with the relative abundance of *Streptococcus* (all q < 0.05, **Figure 4A**).

At the gut microbiota level, MMSE and MoCA scores correlated negatively with the relative abundances of *Streptococcus*, *Raoultella*, *Pseudomonas*, *Enterobacter*, *Klebsiella*, *Staphylococcus*, *Veillonella*, *Fusobacterium* and positively with those of *Butyricicoccus*, *Roseburia*, *Faecalibacterium*, *Ruminococcus* and *Coprococcus* (q < 0.05, **Figure 4B**). HCY levels correlated positively with the relative abundances of *Enterobacter*, *Klebsiella*, and *Staphylococcus*, and negatively with that of *Coprococcus* (q < 0.05, **Figure 4B**). ApoB levels correlated positively with the relative abundances of *Raoultella*, *Veillonella*, *Pseudomonas* and *Enterobacter*, and negatively with that of *Coprococcus* (q < 0.05, **Figure 4B**). ALB levels correlated negatively with the relative abundances of *Enterobacter*, *Klebsiella*, and *Citrobacter*, and positively with those of *Faecalibacterium* and *Ruminococcus* (q < 0.05, **Figure 4B**). ApoA1 levels correlated negatively with the relative abundances of *Enterobacter*, *Klebsiella*, *Raoultella* and *Citrobacter* (q < 0.05, **Figure 4B**).

### 3.5 Predicted functional potential of the oral and gut microbiota in PSCI

**Figure 5:**
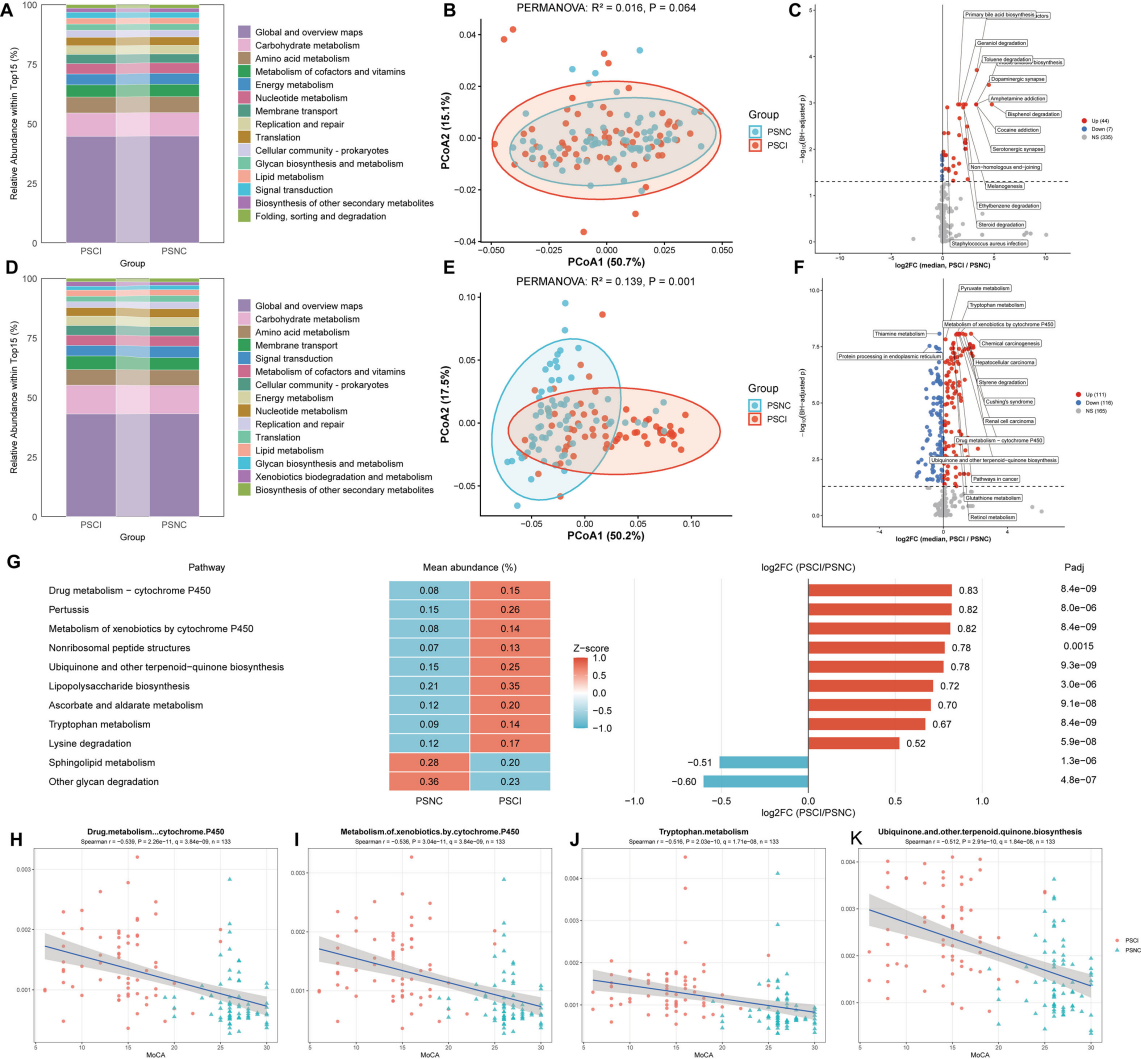
Tax4Fun2-inferred predicted functional potential of the oral and gut microbiota in PSCI versus PSNC. **(A, D)** Stacked bar plots showing the relative abundance of the top 15 Tax4Fun2-inferred predicted KEGG Level 2 pathways in the oral **(A)** and gut **(D)** microbiota of PSCI and PSNC groups. **(B, E)** PCoA based on Bray–Curtis dissimilarity of predicted KEGG Level 3 functional profiles in the oral **(B)** and gut **(E)** microbiota; group differences were assessed using PERMANOVA, with R² and *P* values shown. **(C, F)** Volcano plots summarizing differential predicted KEGG Level 3 pathways between PSCI and PSNC in the oral **(C)** and gut **(F)** microbiota. The x-axis indicates the median log2 fold change (log2FC, PSCI/PSNC), and the y-axis indicates −log10(q value). Pathways are classified as showing higher predicted abundance in PSCI, lower predicted abundance in PSCI, or non-significant according to the q-value threshold. **(G)** Integrated visualization of 11 significantly differential predicted KEGG Level 3 pathways identified after filtering by mean relative abundance > 0.1%, q < 0.05, and |log2FC| > 0.5. The left heatmap shows Z-score-standardized group-averaged predicted pathway abundance in PSCI and PSNC, while the accompanying panel summarizes effect size (log2FC, PSCI/PSNC) and corresponding q values. Red and blue indicate pathways with higher and lower predicted abundance in PSCI, respectively. **(H–K)** Pooled scatter plots showing associations between MoCA scores and representative differential predicted KEGG Level 3 pathway abundance, including **(H)** drug metabolism–cytochrome P450, **(I)** metabolism of xenobiotics by cytochrome P450, **(J)** tryptophan metabolism, and **(K)** ubiquinone and other terpenoid–quinone biosynthesis. Each point represents one participant (red circles, PSCI; blue triangles, PSNC). The solid line indicates the pooled fitted trend, with shaded bands representing 95% confidence intervals. Spearman’s correlation coefficient, P value, BH-adjusted q value, and sample size are annotated in each panel. All functional results represent Tax4Fun2-inferred predicted microbial functional potential rather than direct measurements of metabolic flux or *in vivo* metabolite levels.

Predicted functional profiles of the oral and gut microbiota were inferred from 16S rRNA sequencing data using Tax4Fun2 and the KEGG database. At KEGG Level 2, the predominant functional categories for the oral microbiota were Global and overview maps, Carbohydrate metabolism, Amino acid metabolism, Metabolism of cofactors and vitamins, Energy metabolism, Nucleotide metabolism, Membrane transport, and Replication and repair (**Figure 5A**). Alpha diversity analysis revealed no statistically significant difference in the Shannon index of oral microbiota functions between groups, although a significant difference was observed for the Simpson index (**Supplementary Figures 2A, B**). At KEGG Level 3, PCoA based on Tax4Fun2-inferred oral functional profiles showed only a weak and non-significant trend toward group separation (PERMANOVA: R² = 0.016, *P* = 0.064; **Figure 5B**). However, pathway-level univariate analysis identified 51 differential predicted Level 3 pathways (**Figure 5C**, **Supplementary Table 1**). This should not be interpreted as a contradiction, because PERMANOVA tests whether the overall multivariate functional profile differs between groups, whereas univariate analyses assess each pathway separately.

For the gut microbiota, the main Level 2 functional categories included Global and overview maps, Carbohydrate metabolism, Amino acid metabolism, Membrane transport, Signal transduction, Metabolism of cofactors and vitamins, Cellular community–prokaryotes, and Energy metabolism (**Figure 5D**). Both the Shannon and Simpson indices of gut microbiota functions differed significantly between groups (**Supplementary Figures 2C, D**). PCoA indicated a significant difference in gut microbiota functional structure at Level 3 (PERMANOVA: R² = 0.139, *P* = 0.001; **Figure 5E**). A total of 227 Level 3 pathways were significantly different, with 111 upregulated and 116 downregulated in the PSCI group (**Figure 5F**; **Supplementary Table 2**).

Applying a stringent filter (pathway relative abundance > 0.1%, q value < 0.05, and |logFC| > 0.5) to the differential pathways yielded 11 significantly altered pathways, all from the gut microbiota. These were: Drug metabolism–cytochrome P450, Pertussis, Metabolism of xenobiotics by cytochrome P450, Nonribosomal peptide structures, Ubiquinone and other terpenoid-quinone biosynthesis, Lipopolysaccharide biosynthesis, Ascorbate and aldarate metabolism, Tryptophan metabolism, Lysine degradation, Sphingolipid metabolism, and other glycan degradation (**Figure 5G**; **Supplementary Figure 3A**; **Supplementary Table 3**).

Spearman correlation analysis showed significant associations between these 11 predicted pathways and MMSE/MoCA scores in the pooled cohort (**Supplementary Figure 2E**). In the pooled cohort, four differentially represented Tax4Fun2-predicted KEGG Level 3 pathways met the predefined correlation criteria of q < 0.05 and |r| > 0.5 and were negatively associated with MoCA scores: drug metabolism–cytochrome P450, metabolism of xenobiotics by cytochrome P450, tryptophan metabolism, and ubiquinone and other terpenoid-quinone biosynthesis (**Figures 5H–K**). However, group-stratified Spearman analyses showed that these associations were attenuated and no longer significant within either PSCI or PSNC after FDR correction (**Supplementary Figure 3B**), indicating that the pooled correlations should be interpreted cautiously as exploratory between-group patterns rather than stable within-group associations.

### 3.6 PSCI patients exhibit strengthened oral–gut microbial crosstalk

The oral cavity and intestine, both components of the digestive tract, exhibit bidirectional microbial influences under physiological and pathological conditions. Systematic evidence is lacking, however, on whether PSCI involves enhanced oral–gut microbiota interactions that might participate in or exacerbate PSCI pathogenesis via ectopic colonization or gut oralization. To quantify the closeness of the oral–gut axis in PSCI, we collected paired oral and fecal samples from the same subjects. We then comprehensively assessed oral–gut microecological interactions using three lines of evidence: cross-site shared features, community composition similarity, and the aggregation of oral features in the gut.

**Figure 6:**
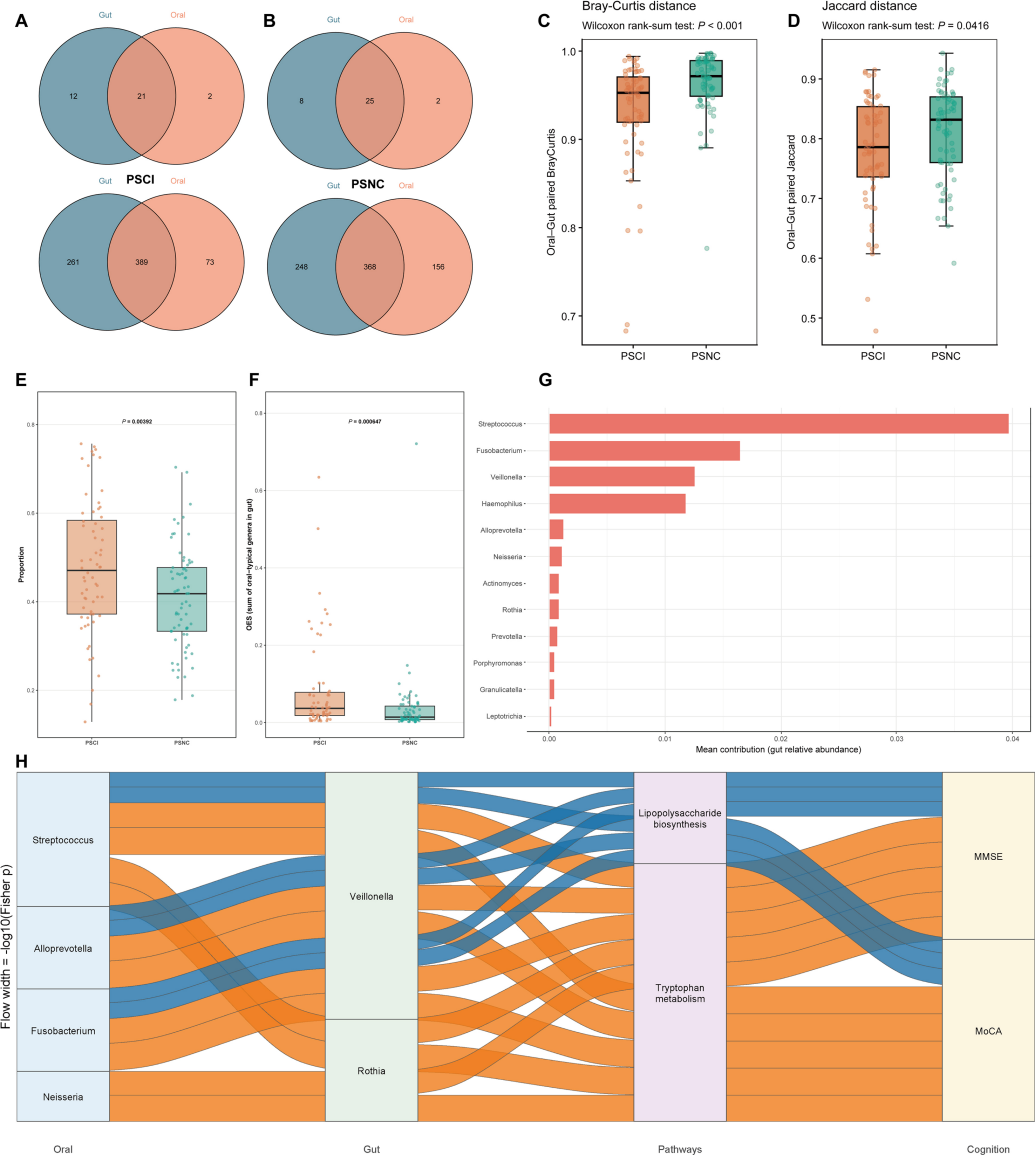
PSCI patients exhibit a stronger gut oralization signal and oral–gut microbial association. **(A, B)** Venn diagrams illustrating the overlap of taxa between the oral and gut microbiota in the PSCI **(A)** and PSNC **(B)** groups. The upper panels summarize the number of overlapping taxa at the phylum level, whereas the lower panels summarize overlaps at the genus level; numbers indicate site-specific taxa and taxa shared between the two sites. **(C, D)** Intra-individual oral–gut community dissimilarity quantified using Bray–Curtis **(C)** and Jaccard **(D)** distances calculated from paired oral and fecal samples. Between-group differences were assessed using the Wilcoxon rank-sum test (BH), with q values shown. **(E)** Comparison of the gut relative abundance contributed by oral–gut shared genera, defined as the summed gut relative abundance of genera detected in both oral and gut samples within each subject. Group differences were evaluated using the Wilcoxon rank-sum test (BH). **(F)** Oral enrichment score (OES), calculated as the summed gut relative abundance of oral-typical genera (defined as genera with oral prevalence >30% and mean oral relative abundance >1%) detected in each subject. Group differences were evaluated using the Wilcoxon rank-sum test (BH). **(G)** Mean contribution (mean gut relative abundance) of each oral-typical genus included in the OES, indicating genera that predominantly drives the oralization signal in the gut. **(H)** Four-layer Sankey diagram integrating significant cross-domain associations among oral genera, gut genera, predicted functional pathways, and cognitive scores. Flow width is proportional to −log10-transformed Fisher’s exact *P* values, reflecting the statistical enrichment of significant associations between adjacent layers, and flows are color-coded to distinguish pathway modules.

At the level of shared microbial features, 21 phyla were shared between the oral and gut communities in the PSCI group, compared with 25 in the PSNC group. The number of shared genera was higher in the PSCI group than in the PSNC group (389 vs. 368), while oral-specific genera were significantly reduced (73 vs. 156) (**Figures 6A, B**). Good’s coverage was uniformly high in both oral and gut samples and did not differ significantly between PSCI and PSNC groups (oral: q = 0.4063; gut: q = 0.9874), suggesting that the shared-genera patterns were unlikely to be driven by post-rarefaction coverage differences (**Supplementary Table 4**). This suggests that microbial features from the oral cavity are more readily detected in the gut of PSCI patients.

Regarding community composition similarity, paired oral–gut microbial dissimilarity was lower in the PSCI group than in the PSNC group. In the unadjusted analysis, PSCI showed a significantly lower paired Bray–Curtis distance than PSNC (median: 0.9527 vs. 0.9716, q < 0.001), with a similar pattern observed for Jaccard distance (median: 0.7858 vs. 0.8317, q = 0.0416; **Figures 6C, D**; **Supplementary Table 5**). Consistently, although a significant site effect was observed in both groups (PERMANOVA, both *P* = 0.001), its explanatory power was lower in PSCI than in PSNC (Bray–Curtis: R² = 0.4396 vs. 0.535; Jaccard: R² = 0.3388 vs. 0.3917; **Supplementary Figure 4**), further suggesting reduced structural divergence between oral and gut microbiota in PSCI. Because Bray–Curtis dissimilarity may be influenced by community evenness, we further performed diversity-adjusted linear regression analyses. The lower paired oral–gut Bray–Curtis distance in PSCI remained significant after adjustment for gut evenness, both gut and oral evenness, and gut Shannon diversity (all *P* ≤ 0.05; **Supplementary Table 6**), indicating that this result was not solely explained by altered microbial evenness. We then constructed quantitative indicators to characterize the aggregation of oral features in the gut. First, the total relative abundance of shared genera in the gut was higher in the PSCI group than in the PSNC group (median: 0.471 vs. 0.418; *P* = 0.00392) (**Figure 6E**). Second, we defined 12 oral-dominant genera (relative abundance >1%, prevalence >30%) and calculated their cumulative abundance in the gut as an Oral Enrichment Score (OES). The OES was significantly higher in the PSCI group (median: 0.0368 vs. 0.0142; *P* = 0.000647) (**Figure 6F**), indicating more prominent gut oralization. Sensitivity analyses using nine combinations of oral mean-abundance and prevalence thresholds showed that OES remained consistently higher in PSCI than in PSNC across all threshold combinations, with all BH-adjusted q values < 0.01 (**Supplementary Table 7**). This supports that the elevated OES in PSCI was not dependent on a single threshold definition. *Streptococcus* was the primary driver of this score, with substantial contributions from *Fusobacterium*, *Veillonella*, and *Haemophilus* (**Figure 6G**; **Supplementary Figure 5A**). Furthermore, among these 12 genera, *Veillonella*, *Granulicatella*, *Rothia*, *Fusobacterium* and *Streptococcus* were significantly elevated in the gut of PSCI patients (q < 0.05) (**Supplementary Figure 5B**), confirming the enrichment of key oral-associated taxa.

Finally, we integrated cross-site correlations of key genera and linked them to predicted gut functional pathways and cognitive scales. Spearman analysis revealed significant correlations among genera present in both sites (**Supplementary Figure 6A**), with some oral-dominant genera showing clustered co-variation with their gut counterparts (**Supplementary Figure 6B**). In PSCI patients, gut *Streptococcus*, *Fusobacterium*, *Granulicatella*, *Veillonella* and *Rothia* correlated with multiple cognitive-related pathways, such as lipopolysaccharide biosynthesis and tryptophan metabolism (**Supplementary Figure 6C**). A multi-layer integrative analysis constructed an association network linking oral genera, gut genera, metabolism pathways, and cognitive scales (MMSE/MoCA). This network indicated that oral *Streptococcus*, *Alloprevotella*, *Fusobacterium*, and *Neisseria* correlated with changes in gut *Neisseria* and *Rothia* and were jointly associated with pathways including lipopolysaccharide (LPS) biosynthesis and tryptophan metabolism, which in turn corresponded to MMSE/MoCA scores (**Figure 6H**).

### 3.7 Exploratory discrimination of PSCI using microbiota-based machine-learning models

Classification models based on six machine learning algorithms were constructed using the relative abundances of oral and gut microbiota at the genus level as features to predict clinical outcomes. The SHAP method was then applied to interpret the model outputs, quantifying the contribution of each bacterial genus to the predictions.

**Figure 7:**
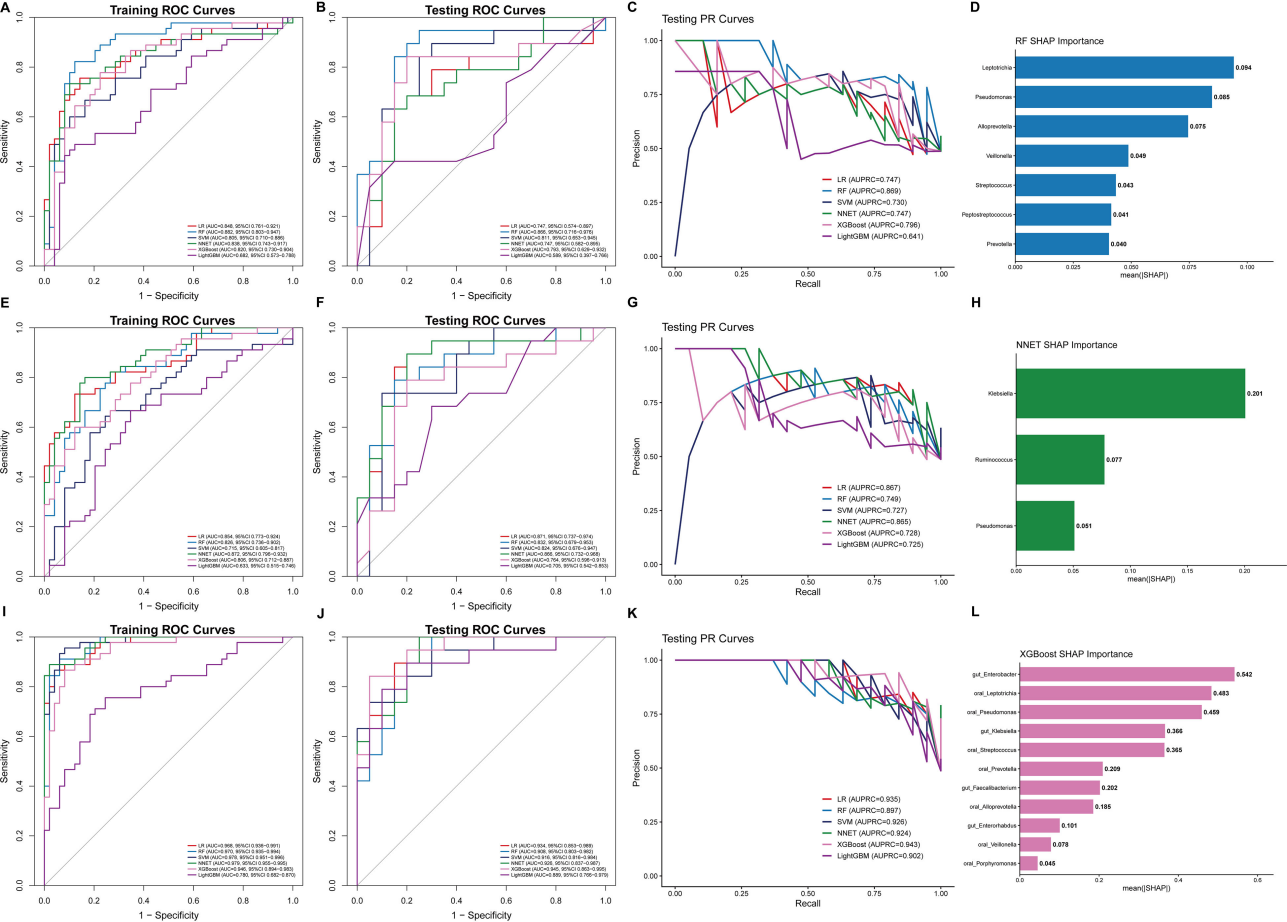
Exploratory machine-learning discrimination of PSCI versus PSNC using oral, gut, and combined oral–gut microbial features. **(A–D)** Performance and interpretability of models built using oral microbiota. **(A)** ROC curves in the train set for six classifiers (LR, RF, SVM, NNET, XGBoost, and LightGBM); AUCs with 95% confidence intervals are provided. **(B)** ROC curves in the test set for the same classifiers. **(C)** PR curves with AP values. **(D)** SHAP-based feature importance (mean absolute SHAP value) for the oral model. **(E–H)** Analogous analyses for models built using gut microbiota: ROC curves in the train set **(E)** and test set **(F)**, PR curves **(G)**, and SHAP feature importance summarizing the most influential gut microbial features **(H)**. **(I–L)** Analogous analyses for models built using combined oral–gut features: ROC curves in the train set **(I)** and test set **(J)**, PR curves **(K)**, and SHAP feature importance **(L)**. In panel **(L)**, features are prefixed with *oral_* or *gut_* to indicate their tissue origin, and bars represent mean(|SHAP|), reflecting each feature’s overall contribution to model predictions. ROC, receiver operating characteristic; PR, precision–recall; AUC, area under the ROC curve; AP, average precision; SHAP, Shapley Additive ExPlanations; LR, logistic regression; RF, random forest; SVM, support vector machine; NNET, neural network; XGBoost, extreme gradient boosting; LightGBM, light gradient boosting machine; CI, confidence interval. The test set was used only for final model evaluation. AUC 95% confidence intervals were estimated by stratified bootstrap resampling. SHAP beeswarm plots showing the direction and distribution of feature effects are provided in **Supplementary Figure 8**.

For the oral microbiota analysis, Boruta feature selection retained 18 candidate bacterial genera (**Supplementary Figure 7A**). LASSO regression was subsequently used for further dimensionality reduction, selecting Lambda.1se (0.05086) as the penalty threshold to identify seven key genera: *Veillonella*, *Streptococcus*, *Prevotella*, *Leptotrichia*, *Alloprevotella*, *Peptostreptococcus*, *Pseudomonas* (**Supplementary Figures 7B, C**). The Random Forest (RF) model demonstrated the best discriminative ability in the train set (AUC = 0.882, 95% CI: 0.803–0.947; **Figure 7A**) and maintained optimal performance in the test set (AUC = 0.866, 95% CI: 0.716–0.976; **Figure 7B**). The PR curve also indicated that the RF model achieved the highest average precision (AP = 0.869), reflecting superior overall performance in identifying positive samples (**Figure 7C**). At the training-derived threshold, the RF model achieved a sensitivity of 0.895, specificity of 0.750, balanced accuracy of 0.822, F1 score of 0.829, Brier score of 0.138, and LogLoss of 0.453 in the test set (**Supplementary Table 8**). SHAP analysis of the RF model revealed that *Leptotrichia* contributed most to the predictions (mean |SHAP| = 0.094), followed by *Pseudomonas* (0.085), *Alloprevotella* (0.075), *Veillonella* (0.075), *Streptococcus* (0.043), *Peptostreptococcus* (0.041), and *Prevotella* (0.040; **Figure 7D**). SHAP beeswarm analysis showed that lower abundances of *Leptotrichia* and *Veillonella*, together with higher abundances of *Streptococcus* and *Prevotella*, tended to shift model predictions toward PSCI (**Supplementary Figure 8A**).

In the gut microbiota analysis, Boruta feature selection retained 13 candidate bacterial genera for subsequent analysis. (**Supplementary Figure 7D**). LASSO regression with Lambda.1se = 0.1007 further identified three key genera: *Klebsiella*, *Ruminococcus* and *Pseudomonas* (**Supplementary Figures 7E, F**). The Neural Network (NNET) model showed the highest discriminative ability in the train set (AUC = 0.872, 95% CI: 0.796–0.932; **Figure 7E**), while the LR model performed best in the test set (AUC = 0.871, 95% CI: 0.737–0.947; **Figure 7F**). The PR curve similarly indicated that LR achieved the highest AP (0.867), suggesting a relative advantage in positive sample identification (**Figure 7G**). At the training-derived threshold, the NNET model achieved a sensitivity of 0.842, specificity of 0.800, balanced accuracy of 0.821, F1 score of 0.821, Brier score of 0.164, and LogLoss of 0.508 in the test set (**Supplementary Table 9**). SHAP analysis based on the NNET model showed that *Klebsiella* contributed most to predictions (mean |SHAP| = 0.201), followed by *Ruminococcus* (0.077) and *Pseudomonas* (0.051; **Figure 7H**). SHAP beeswarm analysis suggested that higher abundances of *Klebsiella* and *Pseudomonas*, together with lower abundance of *Ruminococcus*, tended to shift predictions toward PSCI (**Supplementary Figure 8B**).

For the combined oral and gut microbiota analysis, Boruta feature selection retained 23 candidate bacterial genera for subsequent analysis (**Supplementary Figure 7G**). Subsequent LASSO regression (Lambda.1se = 0.02347) refined the selection to 11 key feature genera: *oral_Veillonella*, *oral_Prevotella*, *oral_Streptococcus*, *oral_Porphyromonas*, *oral_Leptotrichia*, *oral_Alloprevotella*, *oral_Pseudomonas*, *gut_Faecalibacterium*, *gut_Klebsiella*, *gut_Enterobacter*, and *gut_Enterorhabdus* (**Supplementary Figures 7H**, **I**). The NNET model performed best in the train set (AUC = 0.979, 95% CI: 0.955–0.995; **Figure 7I**), while the XGBoost model achieved the highest discriminative ability in the test set (AUC = 0.945, 95% CI: 0.863–0.995; **Figure 7J**). Consistent with the ROC results, the PR curve indicated that XGBoost achieved the highest AP (0.943; **Figure 7K**), demonstrating that combined feature model showed favorable exploratory test-set performance. At the training-derived threshold, the XGBoost model achieved a sensitivity of 0.789, specificity of 0.950, balanced accuracy of 0.870, F1 score of 0.857, Brier score of 0.105, and LogLoss of 0.348 in the test set (**Supplementary Table 10**). SHAP analysis revealed that *gut_Enterobacter* and *oral_Leptotrichia* contributed most to the model output (0.542 and 0.483, respectively), followed by *oral_Pseudomonas* (0.459), *gut_Klebsiella* (0.366), *oral_Streptococcus* (0.365), *oral_Prevotella* (0.209), *gut_Faecalibacterium* (0.202), *oral_Alloprevotella* (0.185), *gut_Enterorhabdus* (0.101), *oral_Veillonella* (0.078), and *oral_Porphyromonas* (0.045; **Figure 7L**). SHAP beeswarm analysis suggested that the higher abundances of *oral_Pseudomonas*, *gut_Klebsiella*, and *oral_Streptococcus*, together with lower abundance of *oral_Leptotrichia*, tended to shift predictions toward PSCI (**Supplementary Figure 8C**).

## 4 Discussion

Both the oral and gut microbiota are established contributors to the pathogenesis of numerous diseases. Consequently, the oral–gut axis concept has emerged to describe the cross-site crosstalk of microorganisms between these compartments and its potential implications for host health and disease (23). Nevertheless, the relationship between the oral microbiota and PSCI, including oral–gut microbial transmission in this context, remains poorly understood. In this study, we performed a comprehensive 16S rRNA sequencing-based analysis of the oral and gut microbiota in patients with PSCI and PSNC. We characterized differences in the diversity and composition of the oral and gut microbiota between these groups and examined their associations with clinical parameters. Functional potential of the microbiota was predicted from the 16S data, revealing differentially abundant pathways between PSCI and PSNC that correlated with cognitive scale performance.

Critically, oral–gut interactions were more pronounced in PSCI patients, as evidenced by a greater number of shared genera, a reduced distance, and a higher OES index. We identified candidate oral-associated genera detectable in the gut and involved in cross-site association patterns. Building on this, we constructed a hierarchical association chain linking “Oral microbiota – Gut microbiota – Metabolic pathways – Cognitive function.” Finally, we developed supervised machine learning models using oral, gut, and combined microbial features to discriminate PSCI from PSNC. The combined oral–gut model showed more favorable exploratory internal discriminative performance than single-site models, suggesting that integrated oral and gut microbial profiles may capture complementary PSCI-associated microbial information.

### 4.1 Microbial alterations in PSCI patients are associated with cognitive status

This study observed a decrease in alpha diversity indices within the oral and gut microbiota of patients with PSCI. In the oral microbiota, the ACE and Chao1 indices were reduced, whereas the Shannon and Simpson indices were decreased in the gut microbiota. These results indicate diminished richness in the oral microbiota and reduced evenness in the gut microbiota of PSCI patients, aligning with prior PSCI research (6, 42). Significant reductions in the Shannon and Simpson indices correlate with various adverse stroke outcomes (43) and are notably altered in neurological disorders such as Alzheimer’s and Parkinson’s diseases (44). Such shifts frequently coincide with inflammatory states, a rise in opportunistic pathogens, and a decline in short-chain fatty acid (SCFA)-producing bacteria (43, 45). Reduced oral alpha diversity similarly signals oral dysbiosis; for example, patients with lung cancer show lower oral Shannon indices (46), and those with Alzheimer’s disease exhibit reduced oral alpha diversity compared to healthy controls (47). This evidence implies that decreased oral microbiome diversity may reflect both diminished local ecological stability and associations with host metabolic disorders and immune dysregulation (48, 49). The direction of change in oral microbiota alpha diversity, however, can vary across diseases, sampling sites, and disease stages (50, 51), necessitating that interpretations account for both sampling site and clinical context.

In the oral cavity, *Pseudomonas*, *Blautia*, *Alloprevotella*, and *Streptococcus* were enriched in PSCI patients, while the relative abundances of genera such as *Leptotrichia* and *Neisseria* were reduced, with all these genera correlating with cognitive function. *Leptotrichia* and *Neisseria* are common commensals in the healthy oral microbiome and are consistently identified in multi-site core microbiome studies (52, 53). In contrast, *Streptococcus* is linked to various oral diseases (54) and may influence cognition via systemic inflammation, endothelial damage, and elevated vascular risk. Specifically, *Streptococcus mutans* strains carrying the collagen-binding protein Cnm are significantly associated with cerebral microbleeds and cognitive impairment, suggesting certain oral streptococcal subtypes may contribute to cerebral small vessel pathology through vascular adhesion or injury (55). An oral dysbiosis characterized by increased *Streptococcus* is also recurrently observed in neurological conditions like Parkinson’s disease, often alongside local oral inflammation (56). Because the present 16S rRNA sequencing analysis was performed at the genus level, these species- or strain-level findings should be considered mechanistic context rather than direct evidence from our dataset. Meanwhile, enrichment of conditionally pathogenic bacteria such as *Pseudomonas* occurs in oral microecology under various disease states, including oral squamous cell carcinoma and mucosal inflammatory diseases (57, 58). A higher colonization load of *Pseudomonas* has also been noted in stroke patients with dysphagia (59). Thus, the oral microbiota in PSCI exhibits a structural imbalance marked by a loss of commensals and an expansion of conditionally pathogenic bacteria.

Within the gut, PSCI patients showed enrichment of Gram-negative bacteria including *Fusobacterium*, *Enterobacter*, *Pseudomonas*, and *Klebsiella*. Conversely, the relative abundances of SCFA-producing bacteria such as *Coprococcus*, *Subdoligranulum*, *Faecalibacterium* and *Roseburia* (60–62) were reduced. Gut *Pseudomonas* is significantly enriched in inflammatory bowel disease and Alzheimer’s disease (63, 64) and correlates with poor outcomes in acute ischemic stroke (65). Similarly, in neurological disorders including stroke, Alzheimer’s disease, and central nervous system infections, the abundance of *Enterobacteriaceae* like *Enterobacter* and *Klebsiella* increases and associates with inflammatory burden and cognitive phenotypes (66–69). SCFAs from microbial fermentation can exert anti-inflammatory and neuroprotective effects by maintaining blood-brain barrier (BBB) integrity and regulating microglial homeostasis (70). A reduction in SCFA producers like *Coprococcus* may lead to SCFA deficiency, thereby impairing barriers and immune homeostasis, amplifying neuroinflammation, and ultimately adversely affecting cognitive outcomes (71). Therefore, the depletion of SCFA-producing genera observed in PSCI may suggest reduced SCFA-producing potential, although SCFA concentrations were not directly measured in this study. These findings raise the hypothesis that gut microecological alterations in PSCI may be associated with immune-metabolic vulnerability related to cognitive impairment.

### 4.2 Tax4Fun2-inferred functional potential suggests pathway-level alterations in PSCI

Tax4Fun2-based functional inference indicated that pathway-level alterations in PSCI were more prominent in gut microbial profiles than in oral microbial profiles. Several predicted pathways, including lipopolysaccharide biosynthesis and tryptophan metabolism, showed associations with cognitive scores in the pooled analysis, suggesting that PSCI may be associated with altered predicted microbial functional potential related to inflammatory and amino acid metabolic pathways. Importantly, Tax4Fun2 infers functional potential from 16S rRNA gene profiles and does not directly measure microbial gene expression, metabolic flux, or *in vivo* metabolite levels. Moreover, group-stratified analyses showed that these pathway–cognition correlations were attenuated and no longer significant within PSCI or PSNC after FDR correction. Therefore, these associations should be interpreted as exploratory pooled patterns that may reflect group-level differences in predicted functional potential rather than stable within-group relationships between pathway abundance and cognitive performance.

An elevated relative abundance of Gram-negative bacteria was observed in both the oral cavity and gut of PSCI patients. In parallel, Tax4Fun2 analysis suggested higher predicted lipopolysaccharide biosynthesis potential in PSCI. LPS, a major structural component of the Gram-negative bacterial outer membrane, can be released during bacterial proliferation, outer membrane vesicle shedding, or cell lysis, thereby acting as an endotoxin to trigger host inflammatory responses (72). Systemically, LPS-induced inflammation can disrupt the BBB, induce structural and functional alterations in astrocytes, promote amyloid and tau pathology, and drive neurodegeneration (73, 74). In both animal and human experimental endotoxin models, LPS exposure elevates inflammatory factors and is linked to cognitive phenotypes such as impaired memory and altered brain network connectivity (75–77). Therefore, the enrichment of Gram-negative taxa together with increased predicted LPS biosynthesis potential may provide a biologically plausible hypothesis linking microbial dysbiosis with inflammation-related pathways and cognitive impairment after stroke. However, because circulating or intestinal LPS levels were not directly measured, these findings should not be interpreted as evidence of increased LPS production or causal LPS-mediated neuroinflammation in PSCI.

Beyond LPS-related functional potential, Tax4Fun2-based analysis also suggested alterations in predicted tryptophan metabolism-related pathways in PSCI. The gut microbiota can directly or indirectly regulate three major tryptophan metabolic routes, including serotonin synthesis, the kynurenine pathway, and the generation of microbially derived indole derivatives (78). These pathways are closely linked to host immune and neuroendocrine regulation and may influence brain function (79, 80). In stroke, kynurenine pathway imbalance has been associated with inflammatory responses, blood–brain barrier dysfunction, and altered balance between neurotoxic and neuroprotective metabolites, which may be relevant to long-term cognitive outcomes (81). Gut microbiota-derived indole derivatives may also act as gut–brain axis mediators through oxidative stress, immune modulation, and neuroinflammatory pathways (82, 83). Experimental evidence further suggests that some indole-related metabolites, such as indole-3-acetic acid and indoxyl sulfate, may regulate microglial inflammatory responses through AhR-related signaling pathways (84–86). These prior studies support the biological relevance of tryptophan metabolism in neuroimmune regulation. Nevertheless, the present study only identified Tax4Fun2-predicted tryptophan metabolism-related functional potential from 16S data and did not directly quantify tryptophan, kynurenine, indole derivatives, or host inflammatory mediators. Thus, these findings should be regarded as hypothesis-generating evidence suggesting a possible microbial functional link to PSCI, requiring validation through metagenomics, metatranscriptomics, targeted metabolomics, inflammatory marker measurements, and experimental studies. Beyond lipopolysaccharide biosynthesis and tryptophan metabolism, the pooled correlation analysis also identified drug metabolism–cytochrome P450, metabolism of xenobiotics by cytochrome P450, and ubiquinone and other terpenoid–quinone biosynthesis as predicted pathways negatively associated with MoCA scores. These pathways may suggest that PSCI-related predicted microbial functional alterations extend to xenobiotic-processing and redox-related processes, which could be relevant to post-stroke medication exposure, oxidative stress, and host–microbe metabolic interactions; however, these Tax4Fun2-based findings remain hypothesis-generating and require validation by metagenomic and metabolomic analyses.

### 4.3 PSCI patients exhibit enhanced oral–gut microbial crosstalk

In this study, we observed stronger oral–gut microbial coupling in PSCI from a cross-ecological perspective. The PSCI group showed a higher number of genera shared between the oral cavity and gut, together with fewer oral-specific genera, suggesting that oral-associated microbial signatures were more readily detected in the gut. Paired within-individual analyses further showed reduced oral–gut community dissimilarity in PSCI, as reflected by lower Bray–Curtis and Jaccard distances, indicating diminished site-specific separation between oral and gut microbial communities. In addition, the PSCI group exhibited a higher cumulative gut abundance of shared genera and an elevated OES, mainly driven by oral-associated taxa such as *Streptococcus*, *Fusobacterium*, *Veillonella*, and *Haemophilus*, supporting a more oral-like gut microbial configuration. However, Bray–Curtis dissimilarity is sensitive to relative abundance distributions and community evenness; therefore, a lower paired oral–gut Bray–Curtis distance should not be interpreted as direct evidence of oral-to-gut microbial transfer by itself. In our diversity-adjusted analyses, the group difference remained significant after adjustment for gut evenness, oral evenness, and gut Shannon diversity, supporting the robustness of reduced oral–gut dissimilarity in PSCI. Nevertheless, this distance-based result was interpreted together with abundance-based OES and shared-genera analyses, rather than as standalone evidence of microbial migration. Accumulating evidence indicates that oral microorganisms are not confined to the oral niche but can enter the digestive tract via swallowing or, under conditions of oral inflammation or barrier impairment, disseminate hematogenous (23, 87). These translocated microbes may become detectable or even colonize the gut, subsequently influencing systemic immune and metabolic homeostasis. For example, periodontitis-associated oral microbiota can induce gut dysbiosis, inhibit intestinal tryptophan metabolism, and exacerbate bone loss in experimental models (88). Specific oral pathogens (e.g., *Porphyromonas*) can translocate to the stomach or intestines, promoting inflammatory responses and contributing to digestive diseases such as chronic atrophic gastritis, ulcerative colitis, and colorectal cancer (18, 89, 90). While *Streptococcus* enrichment is well-established in various oral diseases, oral-origin *Streptococcus anginosus* has been found enriched in the gut of stroke patients and associated with long-term adverse outcomes (91). This provides relevant external evidence that oral-associated taxa may be detectable in the gut after stroke and may be linked to post-stroke outcomes.

The increased detection of oral-associated genera in the gut may reflect not only greater oral microbial exposure but also altered gut ecological resistance. When the structure of the dominant gut microbiota is disrupted, opportunistic pathogens of oral origin may more easily establish themselves and drive immune dysfunction (92). The synchronous dysbiosis of oral and gut microbiota in ischemic stroke patients, accompanied by metabolic disorders, provides external evidence for a stroke-related oral–gut axis (93). In our study, the gut microbiota structure was significantly altered in PSCI patients. Given their more pronounced brain injury and poorer motor function, the observed gut enrichment of oral-associated genera may reflect a disease-related host state characterized by altered oral intake, swallowing function, rehabilitation exposure, and gut ecological resistance. This finding should therefore be interpreted as an associative oral–gut microbial signature rather than evidence of directional microbial transfer or causal involvement in cognitive decline. Recent multi-omics studies integrating oral microbes, the gut microbiome, host metabolome, and disease phenotypes suggest that cross-site microecological interactions can influence host outcomes through metabolic pathways. For instance, combined signatures of plasma proteins, metabolites, and gut microbiota can predict Alzheimer’s disease severity, demonstrating potential clinical utility (94). Periodontitis-associated oral microbiota has been shown to alter the structure of several *Lachnospiraceae* members in the gut during early pregnancy, accompanied by changes in amino acid metabolic pathways and associations with elevated fasting blood glucose (95). Furthermore, during aging, oral and gut microbial communities undergo synergistic remodeling, forming extensive association networks with circulating lipid metabolites, which suggests the oral–gut axis may participate in shaping aging phenotypes by regulating systemic metabolism (96). These findings from diverse disease and population backgrounds provide multidimensional evidence for a pathway linking oral microbiota, gut microbiota, host metabolism, and clinical phenotypes.

In our study, oral-associated genera such as *Streptococcus*, *Alloprevotella*, *Fusobacterium*, and *Neisseria* were detected at higher gut abundances in PSCI patients, supporting a more oral-like gut microbial configuration. Their co-occurrence with gut-detected genera and Tax4Fun2-predicted pathways related to lipopolysaccharide biosynthesis and tryptophan metabolism suggests a possible oral–gut–functional potential–cognition association. However, this pattern should be viewed as hypothesis-generating rather than evidence of directional microbial transfer, altered metabolite production, or causal involvement in PSCI development.

### 4.4 Exploratory discrimination of PSCI using integrated oral–gut microbial features

Using genus-level abundance profiles and training-set-selected microbial features, we constructed supervised machine learning models to distinguish PSCI from PSNC. The combined oral–gut model showed the most favorable exploratory test performance compared with oral-only and gut-only models (AUC: 0.945 vs. 0.866 and 0.871), suggesting that integrating cross-site microbial features may showed higher internal discriminative performance. However, this comparison remains exploratory because multiple algorithms were evaluated without a prespecified primary clinical model and external validation is lacking. This approach is consistent with prior research in other conditions. For colorectal tumors and precancerous lesions, combining oral and fecal microbial features has improved the sensitivity and AUC of classification models beyond those using single-site features (97, 98). Similarly, integrating gut and oral microbial features has enhanced the prediction of vascular complications in type 1 diabetes (99). This pattern extends to neurological phenotypes. In ischemic stroke cohorts, oral and gut microbial features have been used for functional outcome prediction and can be combined with clinical variables to improve performance (100), a strategy also reported in Alzheimer’s disease research (20). In the present study, jointly modeling training-set-selected oral and gut microbial features yielded higher internal discriminatory performance than single-site models. This suggests that combined oral–gut features may more comprehensively capture the systemic microbial signals associated with PSCI. However, our model was evaluated using an internal cohort split into a train set and a test set. An independent external validation cohort has not yet been established. The model’s generalizability and robustness therefore require validation in independent external cohorts, and its real-world application value must be assessed through prospective studies.

### 4.5 Limitations

This study has several limitations. First, its single-center, observational cross-sectional design, while systematically delineating oral and gut microbial differences in PSCI and their associations with cognitive phenotypes, lacks longitudinal follow-up and interventional evidence. Consequently, the temporal sequence and causal direction between microbial alterations and cognitive decline cannot be determined, and findings regarding oral–gut axis interactions in PSCI should be interpreted as associative clues rather than causal evidence. Furthermore, the study population was recruited from a single regional medical center and predominantly comprised hospitalized rehabilitation patients, which may limit generalizability. Although no formal *a priori* sample-size calculation was performed for this exploratory microbiome study, the current sample size provided sufficient sensitivity to detect several moderate-to-large group differences in microbial diversity, oral–gut dissimilarity, and OES; however, it may still be underpowered for detecting weaker associations, subgroup-specific correlations, and small-effect taxa after multiple-testing correction. Therefore, non-significant findings, particularly in stratified correlation and pathway analyses, should be interpreted cautiously. In addition, the sample size remains insufficient to fully capture the clinical heterogeneity of the broader stroke population, including variations in stroke subtype, lesion characteristics, stroke severity, comorbidities, dietary intake, rehabilitation intensity, swallowing function, nutritional status, and oral intake patterns. Notably, detailed dietary variables—such as nutrition route and modifications to food consistency or texture—were not systematically recorded, even though diet is one of the strongest modulators of gut microbiota. Additionally, the 3–6 month post-stroke sampling window may have introduced variability in rehabilitation and swallowing-related factors across participants. Additionally, to ensure feasible cognitive assessments and minimize major confounders, the study excluded individuals unable to complete scale evaluations and those with recent use of microbiota-affecting medications or significant oral/gastrointestinal diseases. While these exclusions enhanced internal consistency, they may also have introduced selection bias. Moreover, outcome stratification relied primarily on screening scales such as the MMSE and MoCA. Although operationally practical, these scales offer limited granularity for specific cognitive domains and can be influenced by education, mood, sleep, fatigue, and comorbidities. Future studies incorporating larger multicenter cohorts, longitudinal sampling, detailed dietary and rehabilitation-related records, systematic neuropsychological assessments, and objective neuroimaging markers would help reduce phenotypic misclassification and strengthen mechanistic interpretation.

Methodologically, the sampling sites for the oral cavity and gut did not comprehensively cover all ecological niches or the mucosa-associated microbiota. Single-time-point sampling also cannot capture the dynamic fluctuations of the microbiota after stroke. Subsequent studies employing multi-site sampling and longitudinal repeated sampling could more accurately characterize temporal changes along the oral–gut axis. Second, the reliance on 16S rRNA amplicon sequencing limits resolution primarily to the genus level, precluding the identification of species/strain-level differences and direct assessment of changes in virulence factors, antibiotic resistance genes, or specific metabolic gene clusters. Functional inferences depend on Tax4Fun2 predictions, which reflect potential functional capacity rather than actual metabolic flux or *in vivo* metabolite levels. Thus, observed correlations between pathways like lipopolysaccharide biosynthesis or tryptophan metabolism and MMSE/MoCA scores should be regarded as testable hypotheses rather than definitive conclusions. Future research integrating metagenomics or metatranscriptomics with metabolomics and concurrent measurement of inflammation- and barrier-related markers would be better suited to construct a closed-loop linking the oral and gut microbiota, metabolic pathways, and the host cognitive phenotype. Furthermore, the enhanced oral–gut interaction proposed here was based on indirect indicators such as community similarity, shared taxa, and the Oral–Gut Ecosystem Similarity index. While these provide quantitative evidence for cross-site coupling, they cannot prove the directionality or causal effect of oral bacterial colonization in the gut. Subsequent confirmation would require strain tracking, culture validation, or source-tracking analysis.

Although we analyzed the laboratory parameters of these patients and identified significant correlations between several clinical indicators and microbial taxa, the current study was unable to further elucidate whether these microbial alterations have functional effects on host metabolic processes. Therefore, future studies integrating multi-omics approaches, including microbiome, metabolomics, and proteomics, together with animal experiments, are needed to clarify the biological significance of these associations and to explore their potential causal mechanisms.

Finally, the machine-learning findings should be interpreted cautiously. Although the pipeline was designed to reduce information leakage by performing train–test splitting before feature selection and by restricting Boruta selection, LASSO regression, model tuning, algorithm comparison, and threshold determination to the train set, the test set remained relatively small. Therefore, the confidence intervals for test-set AUC and AP may still be unstable, and high upper confidence bounds should not be interpreted as evidence of near-perfect generalizability. In addition, because multiple algorithms were compared without a prespecified primary clinical model, the model comparison was exploratory and may be subject to optimism, particularly given the limited sample size and high-dimensional microbiome features. Accordingly, these models should be regarded as showing exploratory internal discriminatory performance rather than representing clinically validated diagnostic tools. Future studies should validate these findings in larger, independent, multicenter prospective cohorts using standardized protocols, with systematic assessment of calibration, decision curve analysis, clinical net benefit, and threshold strategies before clinical translation.

## 5 Conclusion

In this study, we systematically profiled paired oral and gut microbiota in PSCI and performed integrated cross-site analyses to characterize oral–gut microbial ecological patterns. PSCI was associated with microbial remodeling at both sites, including altered diversity, depletion of several commensal taxa, and enrichment of PSCI-associated, opportunistic, or Gram-negative taxa. Tax4Fun2-based inference suggested predicted gut microbial functional potential related to lipopolysaccharide biosynthesis and tryptophan metabolism pathways, which should be interpreted as predicted functional potential rather than direct evidence of metabolic activity. PSCI patients also showed a stronger gut oralization signal, supported by reduced oral–gut dissimilarity, increased gut abundance of shared oral–gut taxa, and elevated OES, although these findings do not prove direct oral-to-gut microbial transfer. Exploratory machine-learning analyses further indicated that combined oral–gut microbial features showed more favorable internal discriminatory performance than single-site models. Larger external, longitudinal, and multi-omics studies are needed to validate these findings and clarify the potential biological mechanisms.

## Data Availability

The raw sequencing data presented in this study are deposited in the NCBI BioProject database, accession numbers PRJNA1471243 and PRJNA1473265.
