# Supplementary material for: Oral and gut microbiota dysbiosis with strengthened oral–gut connectivity in post-stroke cognitive impairment
Source: Front Immunol. 2026 Jun 4;17:1851811. doi: 10.3389/fimmu.2026.1851811 (PMC13276505; doi:10.3389/fimmu.2026.1851811)
Supplement: Supplementary file 1 [file DataSheet1.zip › Supplementary_Material.docx]

Supplementary Material

## Supplementary Figures


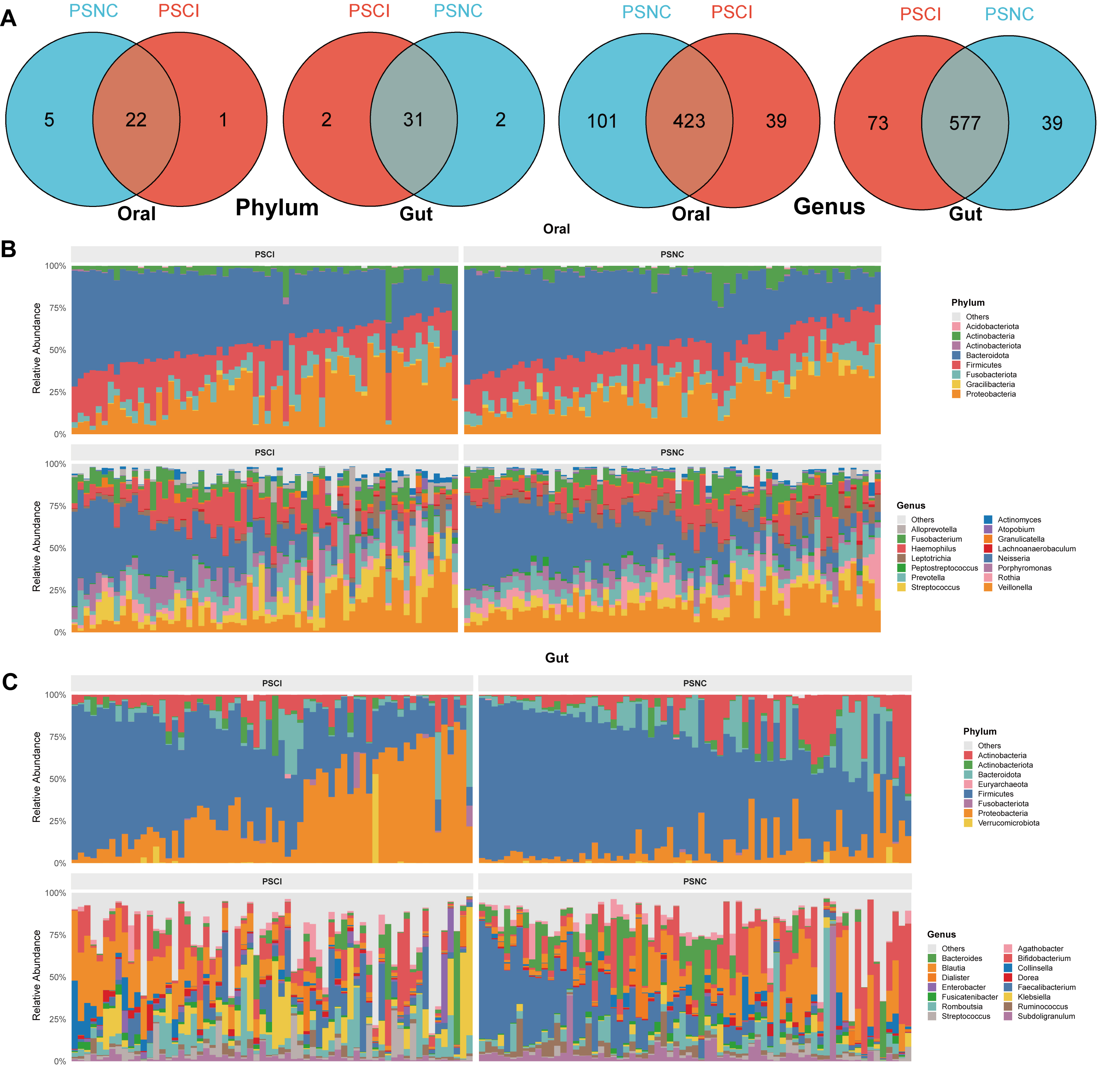


***Supplementary Figure 1. Overall taxonomic profiles and shared taxa between PSCI and PSNC across oral and gut microbiota.***

**(A)** Venn diagrams showing the numbers of taxa shared between PSCI and PSNC and those unique to each group at the phylum and genus levels in the oral and gut microbiota. Numbers indicate the counts of taxa present in each group and their overlap. **(B)** Stacked bar plots depicting the oral microbiota community composition at the phylum (top) and genus (bottom) levels across individuals in the PSCI and PSNC groups. **(C)** Stacked bar plots depicting the gut microbiota community composition at the phylum (top) and genus (bottom) levels across individuals in the PSCI and PSNC groups. In stacked bar plots, each bar represents one subject and colors denote taxa; “Others” indicates low-abundance taxa combined


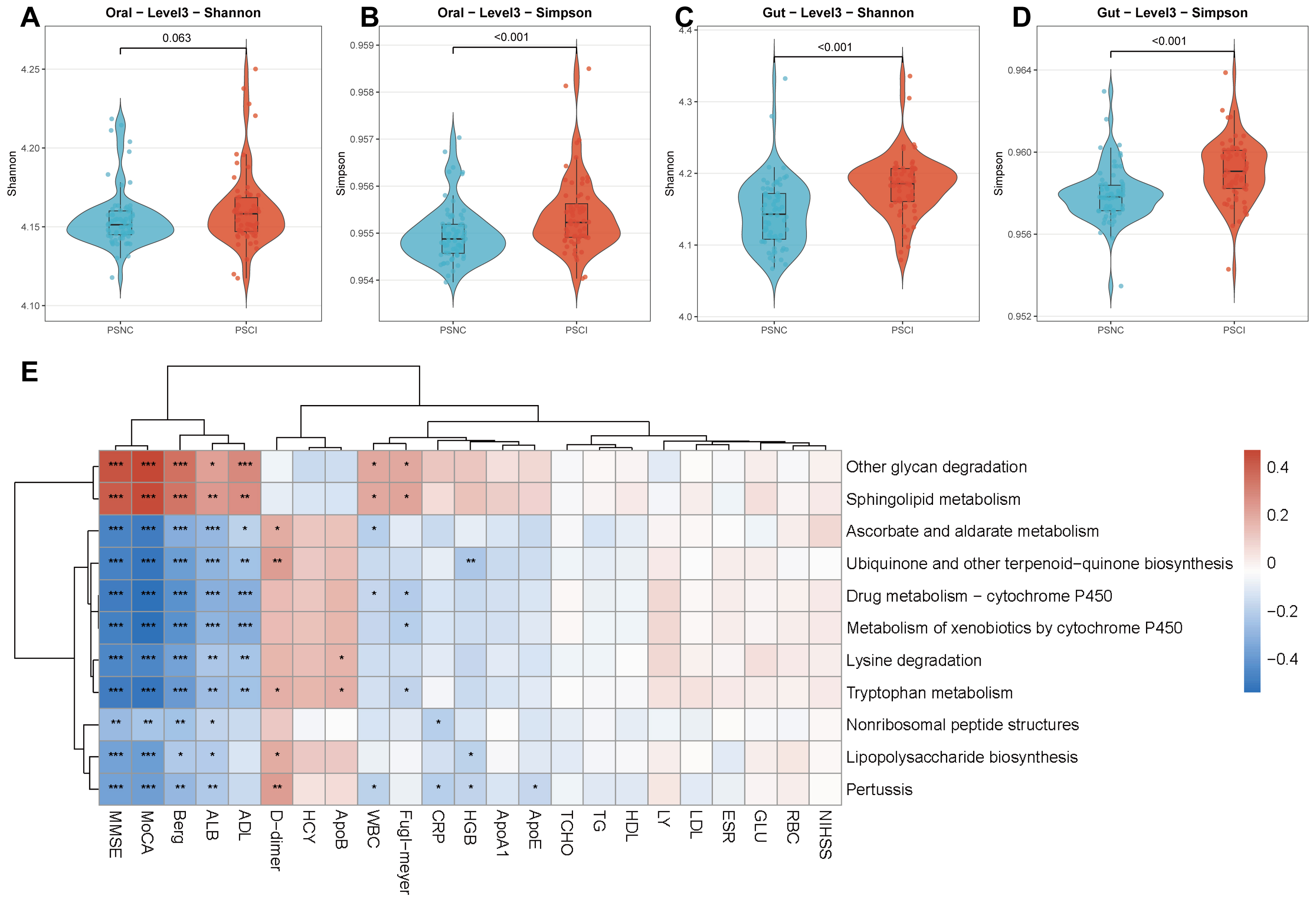


***Supplementary Figure 2. Functional α-diversity of key predicted metabolic pathways and their correlations with clinical variables.***

**(A–D)** Violin/box plots comparing KEGG level 3 functional α-diversity between the PSNC and PSCI groups. Oral functional profiles are shown for Shannon **(A)** and Simpson **(B)** indices, and gut functional profiles are shown for Shannon **(C)** and Simpson **(D)** indices. Between-group differences were evaluated using the Wilcoxon rank-sum test, with P values annotated above each panel.

**(E)** Spearman correlation heatmap depicting associations between filtered PSCI-related predicted KEGG level 3 pathways and clinical/laboratory variables. Colors indicate correlation coefficients (red, positive; blue, negative). Asterisks denote significance levels (* q < 0.05, ** q < 0.01, *** q < 0.001).


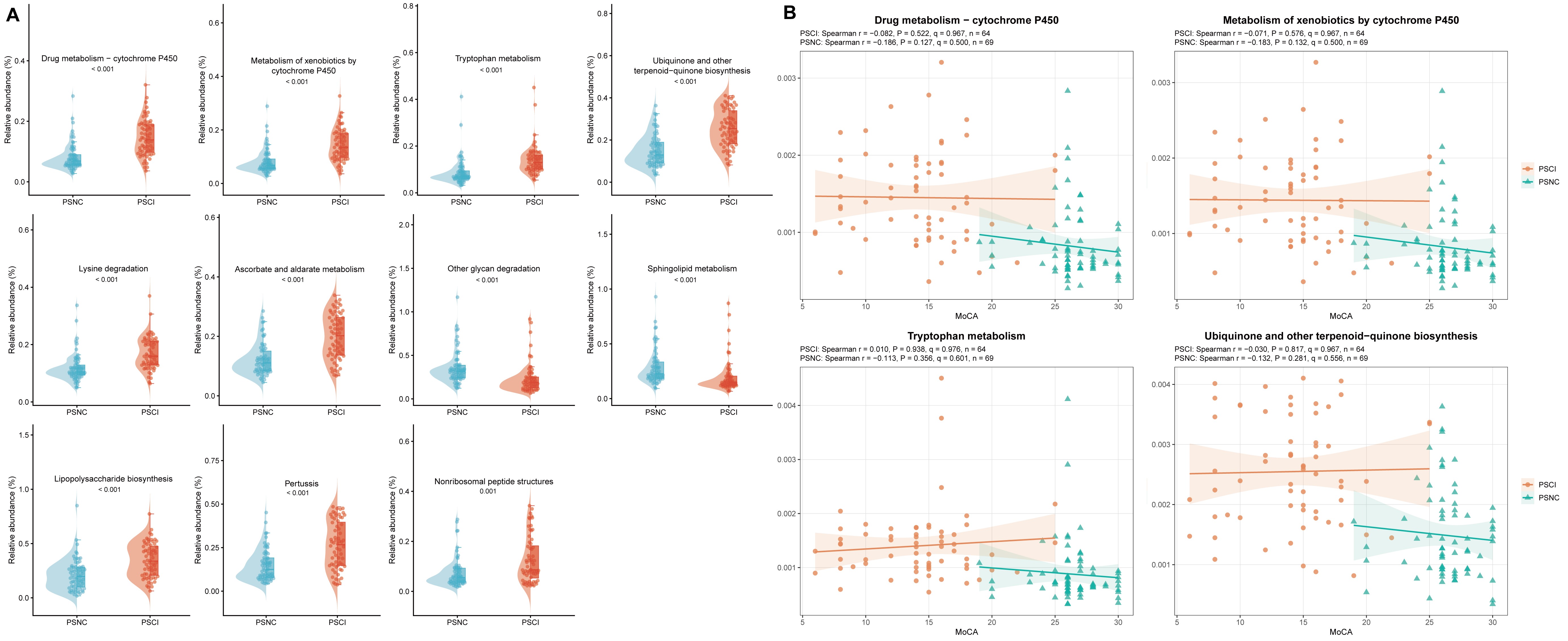


***Supplementary Figure 3. Differential and group-stratified correlation analyses of cognition-related predicted KEGG level 3 pathways.***

(A) Violin plots with overlaid individual points showing the predicted relative abundance (%) of 11 differential KEGG level 3 pathways between PSNC and PSCI. Between-group differences were evaluated using the Wilcoxon rank-sum test, and Benjamini–Hochberg-adjusted *P* values are shown in each panel. (B) Group-stratified Spearman correlation analyses between MoCA scores and four cognition-related predicted KEGG level 3 pathways. Separate fit lines and 95% confidence bands are shown for PSCI and PSNC. Spearman r, nominal *P* values, BH-adjusted q values, and sample sizes are displayed within each panel. PSCI is shown in orange/red, and PSNC is shown in blue/green.

***Supplementary Figure 4. Paired oral–gut ordination reveals site-specific community structure within PSCI and PSNC.***

**(A, B)** PCoA comparing oral and gut microbial communities within the PSCI group based on Bray–Curtis **(A)** and Jaccard **(B)** distances. **(C, D)** Corresponding paired oral–gut PCoA within the PSNC group based on Bray–Curtis **(C)** and Jaccard **(D)** distances. In all panels, each point represents one sample (oral, triangles; gut, circles), and samples from the same participant are connected by dashed lines to indicate paired design. Shaded ellipses denote 95% confidence regions for each site. Site effects (oral vs gut) were tested using PERMANOVA with permutations = 999, stratified/paired by subject; *P* values and explained variance (R²) are shown in each panel.


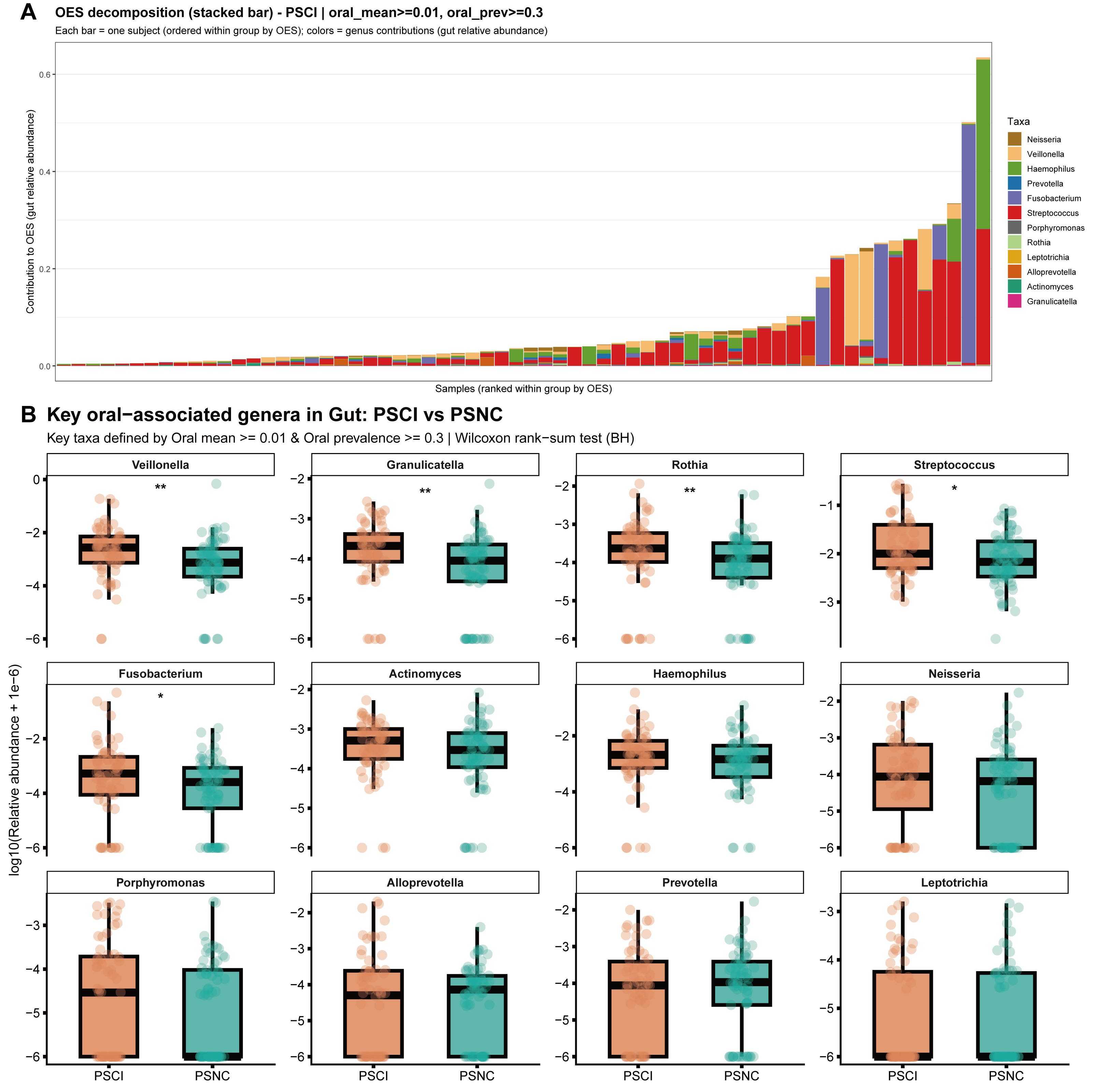


***Supplementary Figure 5. Decomposition of the oral enrichment score (OES) and key oral-associated genera detected in the gut.***

**(A)** Stacked bar plot showing the subject-level decomposition of OES in the PSCI group. Each bar represents one participant (ranked by OES within the group), and the stacked segments indicate the genus-specific contributions to OES, quantified as the gut relative abundance of each oral-associated genus. Oral-associated genera were defined using the thresholds oral mean relative abundance ≥ 0.01 and oral prevalence ≥ 0.3. **(B)** Box/strip plots comparing the gut abundances of key oral-associated genera between PSCI and PSNC. Taxa were defined using the same criteria (oral mean ≥ 0.01 and oral prevalence ≥ 0.3). The y-axis shows log10. Between-group differences were assessed using the Wilcoxon rank-sum test, followed by Benjamini–Hochberg false discovery rate (FDR) correction. ^*^ q < 0.05, ^**^ q < 0.01; OES, oral enrichment score; FDR, false discovery rate; BH, Benjamini–Hochberg.


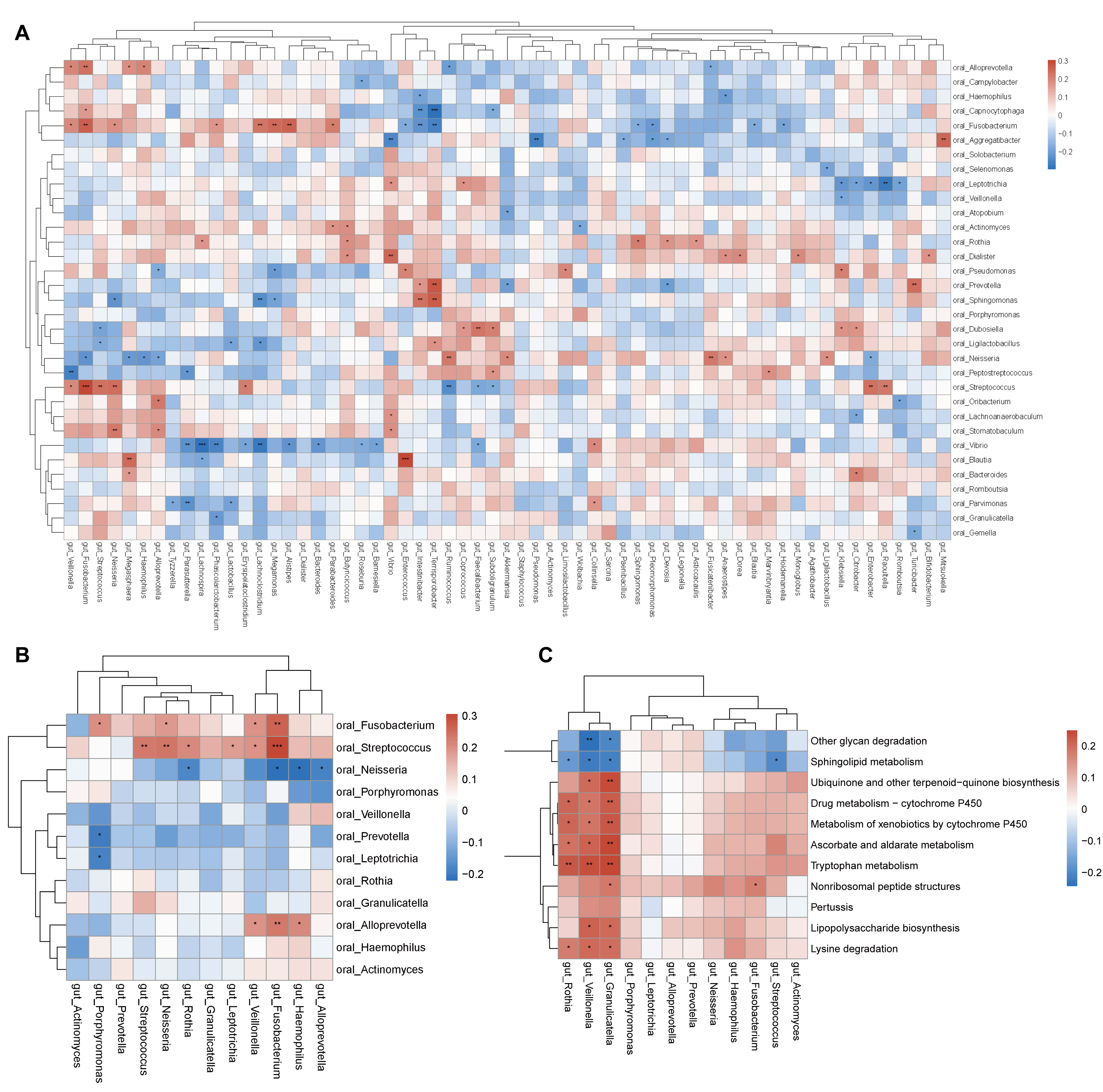


***Supplementary Figure 6. Correlation analyses among oral microbiota, gut microbiota, and predicted metabolic pathways.***

**(A)** Spearman’s rank correlation heatmap depicting pairwise associations between oral genera (rows; prefixed with *oral_*) and gut genera (columns; prefixed with *gut_*). **(B)** Spearman correlation matrix focusing on oral-dominant genera, illustrating their correlation patterns between the oral cavity and the gut. **(C)** Spearman correlation heatmap linking the gut abundances of oral-dominant genera (columns) with PSCI-related predicted KEGG level 3 pathways (rows). Across all panels, colors represent Spearman’s correlation coefficients (ρ; red, positive; blue, negative). Hierarchical clustering (dendrograms) was applied to both axes to visualize correlation-based grouping patterns. Statistical significance is annotated within cells (* q < 0.05, ** q < 0.01, *** q < 0.001).


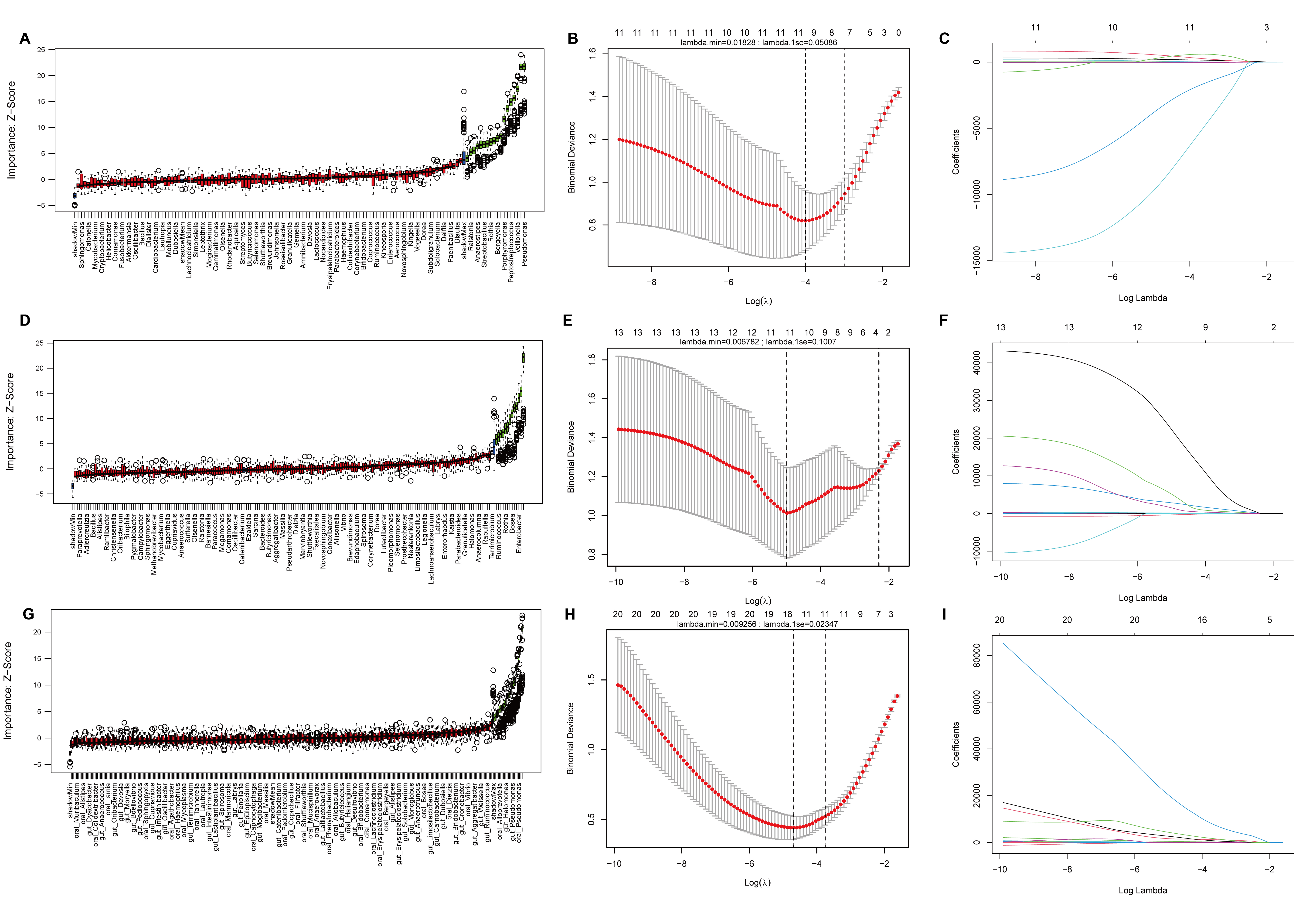


***Supplementary Figure 7. Feature selection of microbial signatures using Boruta and LASSO for oral, gut, and combined oral–gut models.***

(A–C) Feature selection for the oral microbiota model. (A) Boruta feature-screening plot showing the distribution of importance Z-scores across iterations. Shadow features were used as reference variables to identify confirmed microbial features. (B) Ten-fold cross-validation curve of LASSO logistic regression, showing binomial deviance as a function of log(λ). The dashed vertical lines indicate lambda.min = 0.01828 and lambda.1se = 0.05086; the numbers above the plot indicate the number of non-zero coefficients at each λ value. (C) LASSO coefficient paths for oral microbial features as λ changes. (D–F) Feature selection for the gut microbiota model. (D) Boruta importance ranking for gut microbial features. (E) Ten-fold cross-validation curve of LASSO logistic regression with lambda.min = 0.006782 and lambda.1se = 0.1007. (F) Corresponding LASSO coefficient trajectories for gut microbial features. (G–I) Feature selection for the combined oral–gut model. (G) Boruta importance ranking for combined oral and gut microbial features. (H) Ten-fold cross-validation curve of LASSO logistic regression with lambda.min = 0.009256 and lambda.1se = 0.02347. (I) LASSO coefficient paths for the combined feature set, illustrating progressive coefficient shrinkage and sparsity as λ increases. All Boruta and LASSO feature-selection procedures were performed exclusively within the training set to avoid information leakage.


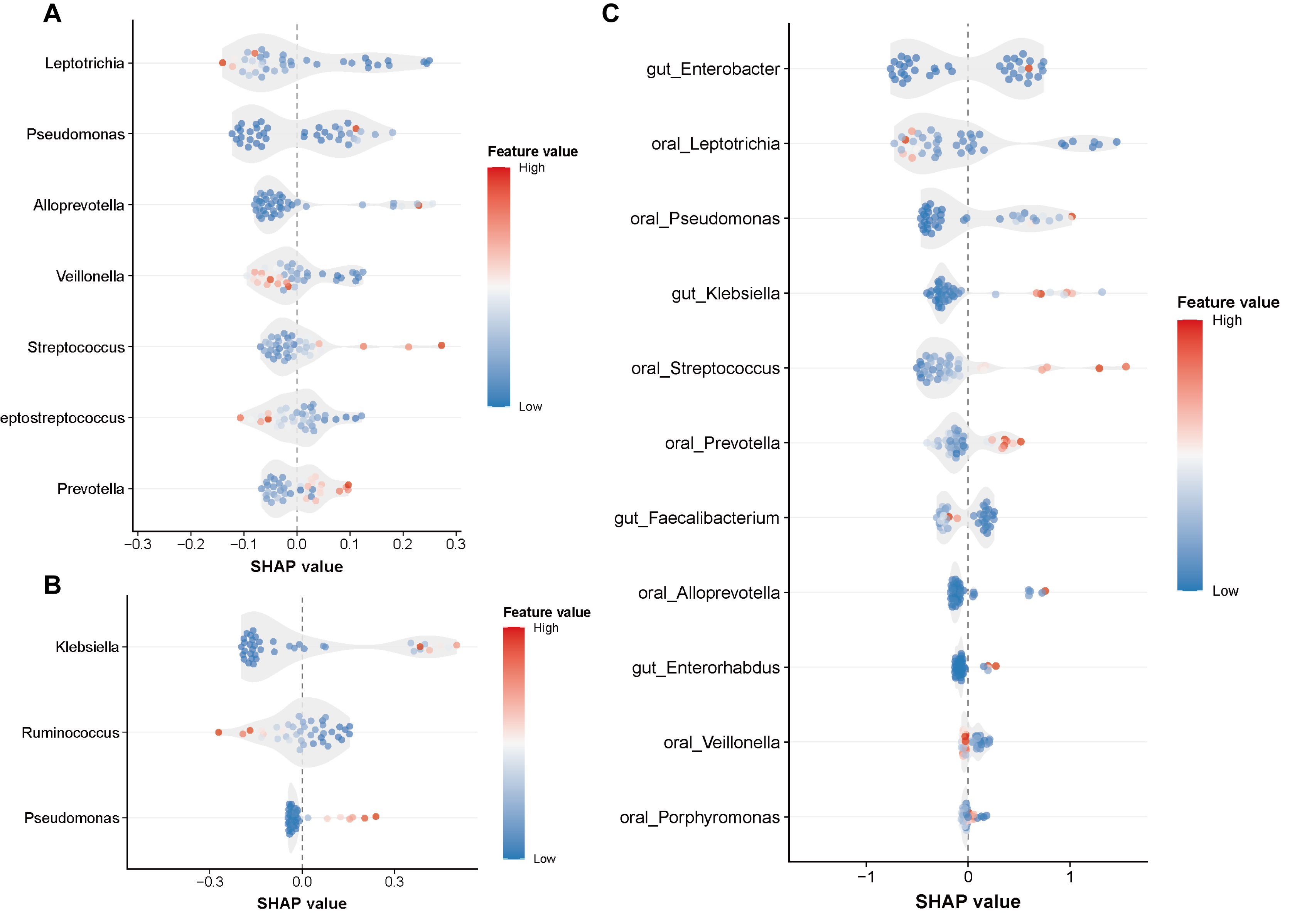


***Supplementary Figure 8. SHAP beeswarm plots for oral, gut, and combined oral–gut machine-learning models.***

(A) SHAP beeswarm plot for the oral microbiota model, showing the direction and magnitude of feature contributions to PSCI classification. (B) SHAP beeswarm plot for the gut microbiota model. (C) SHAP beeswarm plot for the combined oral–gut model, in which oral and gut features are prefixed with oral_ and gut_ to indicate their microbial compartment of origin. Each dot represents one participant. The x-axis indicates the SHAP value, with positive values increasing the model output toward PSCI classification and negative values decreasing it. Feature colors represent the corresponding feature values, with red indicating higher abundance and blue indicating lower abundance. Features are ordered according to their overall contribution to the model.

## Supplementary Tables

***Supplementary Table 1. Predicted KEGG pathways show significant differences in relative abundance in the oral microbiota between the PSCI and PSNC groups.***

| **Pathway** | **log2FC** | ***P*_value** | **q*_value*** | **Status** |
| --- | --- | --- | --- | --- |
| Basal transcription factors | 3.31784 | 0.00000 | 0.00020 | Up |
| Indole alkaloid biosynthesis | 4.49189 | 0.00000 | 0.00041 | Up |
| Amphetamine addiction | 3.26182 | 0.00003 | 0.00108 | Up |
| Bisphenol degradation | 4.78714 | 0.00001 | 0.00108 | Up |
| Cocaine addiction | 3.26182 | 0.00003 | 0.00108 | Up |
| Dopaminergic synapse | 3.26182 | 0.00003 | 0.00108 | Up |
| Geraniol degradation | 1.66967 | 0.00001 | 0.00108 | Up |
| Non-homologous end-joining | 2.03718 | 0.00001 | 0.00108 | Up |
| Primary bile acid biosynthesis | 1.50219 | 0.00002 | 0.00108 | Up |
| Serotonergic synapse | 3.26182 | 0.00003 | 0.00108 | Up |
| Toluene degradation | 2.24131 | 0.00003 | 0.00108 | Up |
| Melanogenesis | 1.99811 | 0.00004 | 0.00125 | Up |
| Staphylococcus aureus infection | 0.44805 | 0.00004 | 0.00125 | Up |
| Steroid degradation | 1.64169 | 0.00008 | 0.00214 | Up |
| Ethylbenzene degradation | 2.38340 | 0.00013 | 0.00324 | Up |
| Neomycin, kanamycin and gentamicin biosynthesis | 0.13287 | 0.00020 | 0.00449 | Up |
| Prolactin signaling pathway | 0.55436 | 0.00019 | 0.00449 | Up |
| Alcoholism | 1.60683 | 0.00023 | 0.00497 | Up |
| Influenza A | 2.22828 | 0.00031 | 0.00624 | Up |
| Neuroactive ligand-receptor interaction | 2.16417 | 0.00037 | 0.00716 | Up |
| Cholesterol metabolism | 2.23589 | 0.00042 | 0.00734 | Up |
| HTLV-I infection | 2.23589 | 0.00040 | 0.00734 | Up |
| Apoptosis - multiple species | 2.21974 | 0.00076 | 0.00981 | Up |
| Colorectal cancer | 2.21974 | 0.00076 | 0.00981 | Up |
| Hepatitis B | 2.21974 | 0.00076 | 0.00981 | Up |
| Herpes simplex infection | 2.20219 | 0.00076 | 0.00981 | Up |
| Human cytomegalovirus infection | 2.22523 | 0.00077 | 0.00981 | Up |
| Kaposi's sarcoma-associated herpesvirus infection | 2.22523 | 0.00077 | 0.00981 | Up |
| Small cell lung cancer | 2.22340 | 0.00079 | 0.00981 | Up |
| Toxoplasmosis | 2.21974 | 0.00077 | 0.00981 | Up |
| Viral myocarditis | 2.21974 | 0.00076 | 0.00981 | Up |
| Two-component system | 0.07000 | 0.00094 | 0.01135 | Up |
| Aminobenzoate degradation | 0.33424 | 0.00120 | 0.01343 | Up |
| Carbon fixation pathways in prokaryotes | -0.05692 | 0.00123 | 0.01343 | Down |
| Quorum sensing | 0.06000 | 0.00121 | 0.01343 | Up |
| Styrene degradation | 0.20318 | 0.00125 | 0.01343 | Up |
| Tropane, piperidine and pyridine alkaloid biosynthesis | -0.05001 | 0.00149 | 0.01552 | Down |
| Secondary bile acid biosynthesis | 1.06532 | 0.00153 | 0.01559 | Up |
| Glycine, serine and threonine metabolism | -0.03476 | 0.00176 | 0.01745 | Down |
| Thyroid hormone signaling pathway | 1.59611 | 0.00212 | 0.02042 | Up |
| Glyoxylate and dicarboxylate metabolism | -0.05867 | 0.00232 | 0.02180 | Down |
| Clavulanic acid biosynthesis | 1.04185 | 0.00250 | 0.02293 | Up |
| Nonribosomal peptide structures | 0.56623 | 0.00302 | 0.02652 | Up |
| Synthesis and degradation of ketone bodies | 0.24839 | 0.00298 | 0.02652 | Up |
| Chlorocyclohexane and chlorobenzene degradation | 0.59517 | 0.00340 | 0.02914 | Up |
| Nitrogen metabolism | -0.04954 | 0.00355 | 0.02977 | Down |
| p53 signaling pathway | 1.27001 | 0.00398 | 0.03268 | Up |
| Ubiquinone and other terpenoid-quinone biosynthesis | -0.05532 | 0.00472 | 0.03793 | Down |
| Furfural degradation | 2.43951 | 0.00576 | 0.04447 | Up |
| Selenocompound metabolism | -0.04251 | 0.00573 | 0.04447 | Down |
| Bile secretion | 1.03084 | 0.00631 | 0.04776 | Up |

Pathway indicates the KEGG pathway name (predicted functional profile).log2FC denotes the log2 fold change of pathway relative abundance (PSCI/PSNC); positive values indicate higher abundance in PSCI, negative values indicate lower abundance in PSCI. *P*_value is the raw two-sided *P* value for between-group comparison. q value is the multiple-testing–adjusted *P* value using the Benjamini–Hochberg (BH) false discovery rate (FDR) procedure. Status summarizes the direction of change (Up = enriched in PSCI; Down = depleted in PSCI), consistent with the sign of log2FC. Pathways shown are those passing the study’s predefined significance threshold based on *Padj_value*.

***Supplementary Table 2. Predicted KEGG pathways showing significant differences in relative abundance in the gut microbiota between the PSCI and PSNC groups.***

| **Pathway** | **log2FC** | ***P*_value** | **q *_value*** | **Status** |
| --- | --- | --- | --- | --- |
| Chemical carcinogenesis | 1.72630 | 0.00000 | 0.00000 | Up |
| Drug metabolism - cytochrome P450 | 1.00513 | 0.00000 | 0.00000 | Up |
| Metabolism of xenobiotics by cytochrome P450 | 0.98887 | 0.00000 | 0.00000 | Up |
| Styrene degradation | 1.20974 | 0.00000 | 0.00000 | Up |
| Thiamine metabolism | -0.24992 | 0.00000 | 0.00000 | Down |
| Tryptophan metabolism | 0.85628 | 0.00000 | 0.00000 | Up |
| Hepatocellular carcinoma | 1.37344 | 0.00000 | 0.00000 | Up |
| Pathways in cancer | 0.79615 | 0.00000 | 0.00000 | Up |
| Ubiquinone and other terpenoid-quinone biosynthesis | 0.97327 | 0.00000 | 0.00000 | Up |
| Pyruvate metabolism | 0.13440 | 0.00000 | 0.00000 | Up |
| Glutathione metabolism | 0.63553 | 0.00000 | 0.00000 | Up |
| Cushing's syndrome | 1.65510 | 0.00000 | 0.00000 | Up |
| Renal cell carcinoma | 1.65510 | 0.00000 | 0.00000 | Up |
| Retinol metabolism | 0.60629 | 0.00000 | 0.00000 | Up |
| Protein processing in endoplasmic reticulum | -0.86559 | 0.00000 | 0.00000 | Down |
| alpha-Linolenic acid metabolism | 1.85732 | 0.00000 | 0.00000 | Up |
| Photosynthesis | -0.46349 | 0.00000 | 0.00000 | Down |
| Ethylbenzene degradation | 1.81061 | 0.00000 | 0.00000 | Up |
| Fluorobenzoate degradation | 1.87651 | 0.00000 | 0.00000 | Up |
| Geraniol degradation | 1.60778 | 0.00000 | 0.00000 | Up |
| Aminobenzoate degradation | 0.60449 | 0.00000 | 0.00000 | Up |
| Insulin resistance | -0.39628 | 0.00000 | 0.00000 | Down |
| Naphthalene degradation | 0.49412 | 0.00000 | 0.00000 | Up |
| Cell cycle - Caulobacter | -0.26370 | 0.00000 | 0.00000 | Down |
| Thyroid hormone synthesis | 0.97370 | 0.00000 | 0.00000 | Up |
| Biosynthesis of siderophore group nonribosomal peptides | 1.34819 | 0.00000 | 0.00000 | Up |
| Toluene degradation | 1.55290 | 0.00000 | 0.00000 | Up |
| MAPK signaling pathway - yeast | 1.20876 | 0.00000 | 0.00000 | Up |
| Lysine degradation | 0.59902 | 0.00000 | 0.00000 | Up |
| Linoleic acid metabolism | 1.74758 | 0.00000 | 0.00000 | Up |
| Longevity regulating pathway | 0.69943 | 0.00000 | 0.00000 | Up |
| Necroptosis | -0.35478 | 0.00000 | 0.00000 | Down |
| One carbon pool by folate | -0.18158 | 0.00000 | 0.00000 | Down |
| Amyotrophic lateral sclerosis (ALS) | 1.09867 | 0.00000 | 0.00000 | Up |
| Bladder cancer | 1.83367 | 0.00000 | 0.00000 | Up |
| Ascorbate and aldarate metabolism | 0.86797 | 0.00000 | 0.00000 | Up |
| FoxO signaling pathway | 0.72017 | 0.00000 | 0.00000 | Up |
| Sulfur metabolism | 0.46961 | 0.00000 | 0.00000 | Up |
| Huntington's disease | 0.73514 | 0.00000 | 0.00000 | Up |
| Cationic antimicrobial peptide (CAMP) resistance | 0.51974 | 0.00000 | 0.00000 | Up |
| Butanoate metabolism | 0.26204 | 0.00000 | 0.00000 | Up |
| Arachidonic acid metabolism | 0.86322 | 0.00000 | 0.00000 | Up |
| Microbial metabolism in diverse environments | 0.13714 | 0.00000 | 0.00000 | Up |
| Zeatin biosynthesis | -0.27684 | 0.00000 | 0.00000 | Down |
| Biosynthesis of unsaturated fatty acids | 0.37623 | 0.00000 | 0.00000 | Up |
| beta-Alanine metabolism | 0.62104 | 0.00000 | 0.00000 | Up |
| Caprolactam degradation | 0.95501 | 0.00000 | 0.00000 | Up |
| Cyanoamino acid metabolism | -0.44049 | 0.00000 | 0.00000 | Down |
| Limonene and pinene degradation | 0.51472 | 0.00000 | 0.00000 | Up |
| Autophagy - yeast | -0.81073 | 0.00000 | 0.00000 | Down |
| Betalain biosynthesis | 0.80265 | 0.00000 | 0.00000 | Up |
| Fatty acid degradation | 0.33300 | 0.00000 | 0.00000 | Up |
| Other glycan degradation | -0.73950 | 0.00000 | 0.00000 | Down |
| Tuberculosis | -0.35676 | 0.00000 | 0.00000 | Down |
| Tyrosine metabolism | 0.25371 | 0.00000 | 0.00000 | Up |
| Prodigiosin biosynthesis | 0.19212 | 0.00000 | 0.00000 | Up |
| Streptomycin biosynthesis | -0.30667 | 0.00000 | 0.00000 | Down |
| DNA replication | -0.32437 | 0.00000 | 0.00000 | Down |
| Glucosinolate biosynthesis | -0.33673 | 0.00000 | 0.00000 | Down |
| Biosynthesis of 12-, 14- and 16-membered macrolides | 1.33390 | 0.00000 | 0.00000 | Up |
| Nucleotide excision repair | -0.36149 | 0.00000 | 0.00000 | Down |
| Histidine metabolism | -0.20230 | 0.00000 | 0.00000 | Down |
| Phenylpropanoid biosynthesis | -0.42627 | 0.00000 | 0.00000 | Down |
| Phosphonate and phosphinate metabolism | 0.39699 | 0.00000 | 0.00000 | Up |
| Proteoglycans in cancer | -0.32633 | 0.00000 | 0.00000 | Down |
| Ether lipid metabolism | 0.97440 | 0.00000 | 0.00000 | Up |
| Glutamatergic synapse | -0.23264 | 0.00000 | 0.00000 | Down |
| Proximal tubule bicarbonate reclamation | 0.63716 | 0.00000 | 0.00000 | Up |
| Alanine, aspartate and glutamate metabolism | -0.13054 | 0.00000 | 0.00000 | Down |
| Propanoate metabolism | 0.17012 | 0.00000 | 0.00000 | Up |
| Protein export | -0.20041 | 0.00000 | 0.00000 | Down |
| Sphingolipid metabolism | -0.62857 | 0.00000 | 0.00000 | Down |
| Amino sugar and nucleotide sugar metabolism | -0.19162 | 0.00000 | 0.00000 | Down |
| Degradation of aromatic compounds | 0.26696 | 0.00000 | 0.00000 | Up |
| RNA degradation | -0.17979 | 0.00000 | 0.00000 | Down |
| Prion diseases | 0.97696 | 0.00000 | 0.00000 | Up |
| MAPK signaling pathway - fly | 0.55605 | 0.00000 | 0.00000 | Up |
| Platinum drug resistance | 0.61679 | 0.00000 | 0.00000 | Up |
| Biotin metabolism | 0.26801 | 0.00000 | 0.00000 | Up |
| Protein digestion and absorption | -1.07536 | 0.00000 | 0.00000 | Down |
| Lipopolysaccharide biosynthesis | 0.77984 | 0.00000 | 0.00000 | Up |
| Chloroalkane and chloroalkene degradation | 0.24276 | 0.00000 | 0.00000 | Up |
| Type I polyketide structures | 0.69596 | 0.00000 | 0.00000 | Up |
| Atrazine degradation | 1.00939 | 0.00000 | 0.00000 | Up |
| Biofilm formation - Escherichia coli | 0.29179 | 0.00000 | 0.00001 | Up |
| Peptidoglycan biosynthesis | -0.28609 | 0.00000 | 0.00001 | Down |
| Benzoate degradation | 0.33217 | 0.00000 | 0.00001 | Up |
| Adipocytokine signaling pathway | -0.59776 | 0.00000 | 0.00001 | Down |
| Basal transcription factors | -1.04966 | 0.00000 | 0.00001 | Down |
| Biosynthesis of amino acids | -0.14430 | 0.00000 | 0.00001 | Down |
| Biosynthesis of type II polyketide products | 1.07766 | 0.00000 | 0.00001 | Up |
| Carbohydrate digestion and absorption | -0.30992 | 0.00000 | 0.00001 | Down |
| Lipoic acid metabolism | 0.57497 | 0.00000 | 0.00001 | Up |
| Ribosome | -0.24688 | 0.00000 | 0.00001 | Down |
| Biosynthesis of vancomycin group antibiotics | -0.34028 | 0.00000 | 0.00001 | Down |
| Carbapenem biosynthesis | -0.26351 | 0.00000 | 0.00001 | Down |
| Type II diabetes mellitus | -0.16902 | 0.00000 | 0.00001 | Down |
| Pertussis | 1.09946 | 0.00000 | 0.00001 | Up |
| Legionellosis | -0.13953 | 0.00000 | 0.00001 | Down |
| Starch and sucrose metabolism | -0.17089 | 0.00000 | 0.00001 | Down |
| Terpenoid backbone biosynthesis | -0.19337 | 0.00000 | 0.00001 | Down |
| Salmonella infection | 0.52497 | 0.00000 | 0.00001 | Up |
| Glycosphingolipid biosynthesis - globo and isoglobo series | -0.62763 | 0.00000 | 0.00001 | Down |
| NOD-like receptor signaling pathway | -0.39258 | 0.00000 | 0.00001 | Down |
| Citrate cycle (TCA cycle) | 0.23797 | 0.00000 | 0.00001 | Up |
| Arginine and proline metabolism | 0.12021 | 0.00000 | 0.00001 | Up |
| Galactose metabolism | -0.11458 | 0.00000 | 0.00001 | Down |
| Phospholipase D signaling pathway | 0.66039 | 0.00000 | 0.00002 | Up |
| Glycosaminoglycan degradation | -0.62511 | 0.00001 | 0.00002 | Down |
| Human papillomavirus infection | -0.16444 | 0.00001 | 0.00002 | Down |
| Lysosome | -0.79365 | 0.00001 | 0.00003 | Down |
| Vancomycin resistance | -0.41289 | 0.00001 | 0.00003 | Down |
| Aminoacyl-tRNA biosynthesis | -0.24284 | 0.00001 | 0.00003 | Down |
| Flavone and flavonol biosynthesis | -0.71947 | 0.00001 | 0.00003 | Down |
| Two-component system | -0.23476 | 0.00001 | 0.00003 | Down |
| PPAR signaling pathway | -0.33013 | 0.00001 | 0.00003 | Down |
| Nitrogen metabolism | 0.18465 | 0.00001 | 0.00003 | Up |
| Viral carcinogenesis | -0.16829 | 0.00001 | 0.00005 | Down |
| Pyrimidine metabolism | -0.08651 | 0.00002 | 0.00007 | Down |
| Oxidative phosphorylation | 0.08660 | 0.00002 | 0.00007 | Up |
| Mismatch repair | -0.18650 | 0.00002 | 0.00008 | Down |
| Biofilm formation - Vibrio cholerae | 0.30489 | 0.00002 | 0.00008 | Up |
| Metabolic pathways | -0.02282 | 0.00003 | 0.00009 | Down |
| ABC transporters | 0.07085 | 0.00003 | 0.00010 | Up |
| Phosphatidylinositol signaling system | 0.11873 | 0.00003 | 0.00010 | Up |
| Amoebiasis | -1.02455 | 0.00004 | 0.00012 | Down |
| Lysine biosynthesis | -0.12681 | 0.00004 | 0.00012 | Down |
| Longevity regulating pathway - worm | -0.15004 | 0.00005 | 0.00014 | Down |
| Glycosphingolipid biosynthesis - ganglio series | -0.86706 | 0.00006 | 0.00017 | Down |
| Biosynthesis of enediyne antibiotics | 0.99447 | 0.00006 | 0.00018 | Up |
| Valine, leucine and isoleucine degradation | 0.16180 | 0.00006 | 0.00018 | Up |
| Homologous recombination | -0.13119 | 0.00007 | 0.00019 | Down |
| Nitrotoluene degradation | 0.49825 | 0.00007 | 0.00019 | Up |
| Xylene degradation | 1.11738 | 0.00009 | 0.00025 | Up |
| Various types of N-glycan biosynthesis | -0.82659 | 0.00009 | 0.00026 | Down |
| Neomycin, kanamycin and gentamicin biosynthesis | -0.22825 | 0.00010 | 0.00028 | Down |
| Purine metabolism | -0.03682 | 0.00012 | 0.00035 | Down |
| Vibrio cholerae infection | 1.52903 | 0.00012 | 0.00035 | Up |
| Proteasome | -0.98593 | 0.00015 | 0.00041 | Down |
| Biosynthesis of secondary metabolites | -0.03572 | 0.00016 | 0.00045 | Down |
| Isoflavonoid biosynthesis | -1.04067 | 0.00017 | 0.00047 | Down |
| Polycyclic aromatic hydrocarbon degradation | 0.98895 | 0.00017 | 0.00047 | Up |
| Polyketide sugar unit biosynthesis | -0.19012 | 0.00019 | 0.00052 | Down |
| Pentose phosphate pathway | 0.09534 | 0.00021 | 0.00058 | Up |
| RIG-I-like receptor signaling pathway | 1.05695 | 0.00023 | 0.00063 | Up |
| Acarbose and validamycin biosynthesis | -0.20849 | 0.00025 | 0.00068 | Down |
| Ferroptosis | -0.36424 | 0.00026 | 0.00070 | Down |
| Glucagon signaling pathway | -0.18980 | 0.00027 | 0.00071 | Down |
| Bacterial invasion of epithelial cells | 0.61235 | 0.00030 | 0.00078 | Up |
| Porphyrin and chlorophyll metabolism | -0.19937 | 0.00030 | 0.00079 | Down |
| Phosphotransferase system (PTS) | 0.34169 | 0.00032 | 0.00083 | Up |
| Bacterial secretion system | 0.18597 | 0.00033 | 0.00086 | Up |
| Glycosaminoglycan biosynthesis - heparan sulfate / heparin | -1.28900 | 0.00040 | 0.00103 | Down |
| Quorum sensing | -0.12650 | 0.00040 | 0.00103 | Down |
| Pathogenic Escherichia coli infection | 2.15661 | 0.00042 | 0.00107 | Up |
| Thermogenesis | -0.31918 | 0.00044 | 0.00111 | Down |
| Folate biosynthesis | 0.09927 | 0.00045 | 0.00111 | Up |
| Taurine and hypotaurine metabolism | 0.11598 | 0.00045 | 0.00111 | Up |
| Caffeine metabolism | 1.37586 | 0.00051 | 0.00126 | Up |
| Base excision repair | -0.09396 | 0.00055 | 0.00134 | Down |
| Nonribosomal peptide structures | 0.65652 | 0.00062 | 0.00150 | Up |
| D-Glutamine and D-glutamate metabolism | -0.08121 | 0.00066 | 0.00159 | Down |
| Antifolate resistance | -0.12353 | 0.00067 | 0.00161 | Down |
| Primary bile acid biosynthesis | -0.54609 | 0.00074 | 0.00176 | Down |
| Glycosphingolipid biosynthesis - lacto and neolacto series | -0.78341 | 0.00079 | 0.00187 | Down |
| Riboflavin metabolism | -0.12844 | 0.00080 | 0.00189 | Down |
| Dioxin degradation | 0.79469 | 0.00093 | 0.00217 | Up |
| Fructose and mannose metabolism | 0.11705 | 0.00094 | 0.00220 | Up |
| PI3K-Akt signaling pathway | -0.37648 | 0.00097 | 0.00225 | Down |
| Estrogen signaling pathway | -0.34057 | 0.00104 | 0.00239 | Down |
| Prolactin signaling pathway | -0.28462 | 0.00110 | 0.00253 | Down |
| Phenylalanine, tyrosine and tryptophan biosynthesis | -0.13998 | 0.00112 | 0.00256 | Down |
| Apoptosis - fly | 0.23452 | 0.00114 | 0.00257 | Up |
| Staurosporine biosynthesis | 1.50481 | 0.00114 | 0.00257 | Up |
| N-Glycan biosynthesis | -0.70431 | 0.00121 | 0.00272 | Down |
| RNA polymerase | -0.12880 | 0.00123 | 0.00275 | Down |
| Biosynthesis of type II polyketide backbone | 1.22595 | 0.00144 | 0.00319 | Up |
| Glycosaminoglycan biosynthesis - chondroitin sulfate / dermatan sulfate | -1.37027 | 0.00151 | 0.00333 | Down |
| Methane metabolism | -0.03897 | 0.00153 | 0.00336 | Down |
| IL-17 signaling pathway | -0.33170 | 0.00158 | 0.00339 | Down |
| Progesterone-mediated oocyte maturation | -0.33170 | 0.00158 | 0.00339 | Down |
| Prostate cancer | -0.33170 | 0.00158 | 0.00339 | Down |
| Th17 cell differentiation | -0.33170 | 0.00158 | 0.00339 | Down |
| Antigen processing and presentation | -0.33147 | 0.00161 | 0.00343 | Down |
| Carbon fixation in photosynthetic organisms | -0.04667 | 0.00187 | 0.00397 | Down |
| Chronic myeloid leukemia | -1.56608 | 0.00205 | 0.00430 | Down |
| Wnt signaling pathway | -1.56608 | 0.00205 | 0.00430 | Down |
| Drug metabolism - other enzymes | 0.04291 | 0.00235 | 0.00490 | Up |
| Cholesterol metabolism | -0.65971 | 0.00242 | 0.00502 | Down |
| Notch signaling pathway | -1.11693 | 0.00261 | 0.00538 | Down |
| MAPK signaling pathway - plant | 0.28327 | 0.00316 | 0.00648 | Up |
| HTLV-I infection | -0.65054 | 0.00320 | 0.00654 | Down |
| Flagellar assembly | 0.76550 | 0.00365 | 0.00742 | Up |
| Apoptosis | -0.69295 | 0.00440 | 0.00888 | Down |
| HIF-1 signaling pathway | -0.11129 | 0.00452 | 0.00909 | Down |
| 2-Oxocarboxylic acid metabolism | -0.07060 | 0.00506 | 0.01012 | Down |
| Neuroactive ligand-receptor interaction | -0.59307 | 0.00542 | 0.01079 | Down |
| Vitamin B6 metabolism | 0.23656 | 0.00558 | 0.01104 | Up |
| Transcriptional misregulation in cancer | -1.51951 | 0.00597 | 0.01177 | Down |
| Meiosis - yeast | -0.66623 | 0.00614 | 0.01203 | Down |
| Choline metabolism in cancer | 0.26664 | 0.00704 | 0.01372 | Up |
| Rheumatoid arthritis | -1.63237 | 0.00713 | 0.01384 | Down |
| Hippo signaling pathway - fly | 1.29295 | 0.00728 | 0.01398 | Up |
| Hippo signaling pathway - multiple species | 1.29295 | 0.00728 | 0.01398 | Up |
| Bile secretion | 1.03242 | 0.00742 | 0.01420 | Up |
| Shigellosis | 1.55932 | 0.00753 | 0.01432 | Up |
| Insulin signaling pathway | -0.14673 | 0.00849 | 0.01607 | Down |
| Glycerophospholipid metabolism | 0.05917 | 0.00918 | 0.01731 | Up |
| Secondary bile acid biosynthesis | -0.38185 | 0.00968 | 0.01815 | Down |
| Osteoclast differentiation | -1.66625 | 0.00981 | 0.01830 | Down |
| Retrograde endocannabinoid signaling | 0.78650 | 0.01020 | 0.01894 | Up |
| GABAergic synapse | -0.10359 | 0.01160 | 0.02145 | Down |
| RNA transport | -0.29615 | 0.01268 | 0.02334 | Down |
| Carbon metabolism | 0.02488 | 0.01317 | 0.02391 | Up |
| Dilated cardiomyopathy (DCM) | -0.82145 | 0.01317 | 0.02391 | Down |
| Synthesis and degradation of ketone bodies | 0.26864 | 0.01317 | 0.02391 | Up |
| AGE-RAGE signaling pathway in diabetic complications | -1.00811 | 0.01403 | 0.02500 | Down |
| Hypertrophic cardiomyopathy (HCM) | -0.67320 | 0.01403 | 0.02500 | Down |
| Platelet activation | -1.00811 | 0.01403 | 0.02500 | Down |
| Relaxin signaling pathway | -1.00811 | 0.01403 | 0.02500 | Down |
| Penicillin and cephalosporin biosynthesis | -0.33545 | 0.01609 | 0.02853 | Down |
| Novobiocin biosynthesis | -0.05138 | 0.01669 | 0.02947 | Down |
| Chagas disease (American trypanosomiasis) | 0.73462 | 0.02203 | 0.03872 | Up |
| Fatty acid metabolism | 0.06048 | 0.02256 | 0.03947 | Up |
| Glyoxylate and dicarboxylate metabolism | 0.08001 | 0.02282 | 0.03976 | Up |
| Phenazine biosynthesis | 0.09034 | 0.02687 | 0.04661 | Up |
| Clavulanic acid biosynthesis | 0.81323 | 0.02846 | 0.04916 | Up |

Pathway denotes the predicted KEGG level 3 functional pathway. log2FC represents the log2 fold change in pathway relative abundance (PSCI/PSNC); log2FC > 0 indicates enrichment in PSCI, whereas log2FC < 0 indicates depletion in PSCI. *P*_value is the raw two-sided *P* value for the between-group comparison. q value is the multiple-testing–adjusted *P* value using the Benjamini–Hochberg (BH) false discovery rate (FDR) procedure. Status summarizes the direction of change (Up, higher in PSCI; Down, lower in PSCI), consistent with the sign of log2FC.

***Supplementary Table 3. Eleven PSCI-associated differential predicted KEGG level 3 pathways after abundance and effect-size filtering.***

| **Pathway** | **log2FC** | ***P*_value** | **q_value** |
| --- | --- | --- | --- |
| Drug metabolism - cytochrome P450 | 0.82538 | < 0.001 | < 0.001 |
| Pertussis | 0.82409 | < 0.001 | < 0.001 |
| Metabolism of xenobiotics by cytochrome P450 | 0.81645 | < 0.001 | < 0.001 |
| Nonribosomal peptide structures | 0.78462 | < 0.001 | 0.002 |
| Ubiquinone and other terpenoid-quinone biosynthesis | 0.77760 | < 0.001 | < 0.001 |
| Lipopolysaccharide biosynthesis | 0.71793 | < 0.001 | < 0.001 |
| Ascorbate and aldarate metabolism | 0.70439 | < 0.001 | < 0.001 |
| Tryptophan metabolism | 0.67292 | < 0.001 | < 0.001 |
| Lysine degradation | 0.52378 | < 0.001 | < 0.001 |
| Sphingolipid metabolism | -0.51044 | < 0.001 | < 0.001 |
| Other glycan degradation | -0.60233 | < 0.001 | < 0.001 |

Pathway indicates the KEGG level 3 predicted functional pathway. log2FC denotes the log2 fold change of pathway relative abundance (PSCI/PSNC); positive values indicate enrichment in PSCI, whereas negative values indicate depletion in PSCI. *P*_value is the raw two-sided *P* value for between-group comparison. q_value is the Benjamini–Hochberg (BH) FDR–adjusted *P* value. Pathways listed are those meeting the predefined screening criteria (mean relative abundance > 0.1%, *Padj* value < 0.05, and |log2FC| > 0.5).

***Supplementary Table 4. Good’s coverage statistics by group and sample type***

| **Sample type** | **Group** | **n** | **Good’s coverage**  **mean ± SD** | **Good’s coverage**  **median (IQR)** | **Range** | **Wilcoxon *P* value** | **BH-adjusted q value** |
| --- | --- | --- | --- | --- | --- | --- | --- |
| Oral | PSCI | 64 | 0.9967 ± 0.0008 | 0.9969 (0.9965–0.9972) | 0.9936–0.9980 | 0.2032 | 0.4063 |
| Oral | PSNC | 69 | 0.9967 ± 0.0007 | 0.9967 (0.9964–0.9971) | 0.9936–0.9979 |  |  |
| Gut | PSCI | 64 | 0.9981 ± 0.0009 | 0.9983 (0.9980–0.9986) | 0.9933–0.9990 | 0.9874 | 0.9874 |
| Gut | PSNC | 69 | 0.9981 ± 0.0009 | 0.9983 (0.9980–0.9986) | 0.9931–0.9993 |  |  |

Good’s coverage values are presented as mean ± SD and median (IQR). Between-group comparisons were performed using the Wilcoxon rank-sum test. P values were adjusted using the Benjamini–Hochberg method across oral and gut comparisons.

***Supplementary Table 5. Original paired oral–gut distance comparison***

| **Distance metric** | **Group** | **n** | **Mean ± SD** | **Median (IQR)** | **Range** | **Wilcoxon**  ***P* value** | **BH-adjusted**  **q value** |
| --- | --- | --- | --- | --- | --- | --- | --- |
| Bray-Curtis | PSCI | 64 | 0.9311 ± 0.0633 | 0.9527 (0.9194–0.9707) | 0.6829–0.9940 | < 0.001 | < 0.001 |
| Bray-Curtis | PSNC | 69 | 0.9649 ± 0.0349 | 0.9716 (0.9488–0.9892) | 0.7765–0.9976 |  |  |
| Jaccard | PSCI | 64 | 0.7788 ± 0.0944 | 0.7858 (0.7360–0.8535) | 0.4783–0.9152 | 0.0416 | 0.0416 |
| Jaccard | PSNC | 69 | 0.8118 ± 0.0771 | 0.8317 (0.7600–0.8699) | 0.5917–0.9431 |  |  |

Values are presented as mean ± SD, median (IQR), and range. Between-group comparisons were performed using the Wilcoxon rank-sum test. P values were adjusted using the Benjamini–Hochberg method across the two-distance metrics. Lower values indicate reduced paired oral–gut community dissimilarity.

***Supplementary Table 6. Diversity-adjusted analysis of paired oral–gut Bray-Curtis dissimilarity***

| **Distance metric** | **Model** | **Adjustment variables** | **β for PSCI vs PSNC** | **95% CI** | ***P* value** | **R²** | **Interpretation** |
| --- | --- | --- | --- | --- | --- | --- | --- |
| Bray-Curtis | Unadjusted | None | -0.0338 | -0.0512 to -0.0164 | <0.001 | 0.102 | PSCI lower |
| Bray-Curtis | Adjusted model 1 | Gut evenness | -0.0311 | -0.0487 to -0.0135 | <0.001 | 0.120 | PSCI lower |
| Bray-Curtis | Adjusted model 2 | Gut evenness + oral evenness | -0.0330 | -0.0504 to -0.0156 | <0.001 | 0.156 | PSCI lower |
| Bray-Curtis | Adjusted model 3 | Gut Shannon | -0.0294 | -0.0467 to -0.0121 | 0.001 | 0.147 | PSCI lower |
| Jaccard | Unadjusted | None | -0.0330 | -0.0625 to -0.0036 | 0.028 | 0.036 | PSCI lower |
| Jaccard | Adjusted model | Gut evenness | -0.0379 | -0.0676 to -0.0081 | 0.013 | 0.058 | PSCI lower |

β represents the estimated difference in paired oral–gut distance for PSCI relative to PSNC, with PSNC used as the reference group. Negative β values indicate lower paired oral–gut dissimilarity in PSCI. Linear regression models were used for diversity-adjusted analyses. Gut evenness was included as the primary adjustment variable because Bray–Curtis dissimilarity is abundance-weighted and may be influenced by community evenness. Oral evenness and gut Shannon diversity were included as complementary α-diversity covariates to assess the robustness of the group effect. R² represents the proportion of variance explained by the entire model. Bray–Curtis distance reflects abundance-weighted community dissimilarity, whereas Jaccard distance reflects presence/absence-based community dissimilarity.

***Supplementary Table 7. Sensitivity analysis of the Oral Enrichment Score under different oral-abundance and oral-prevalence thresholds***

| **Oral mean abundance cutoff** | **Oral prevalence cutoff** | **Number of selected genera** | **PSCI OES, median IQR,**  **n = 64** | **PSNC OES, median IQR,**  **n = 69** | **Wilcoxon *P* value** | **BH-adjusted q value** | **Direction** |
| --- | --- | --- | --- | --- | --- | --- | --- |
| ≥0.5% | ≥20% | 15 | 0.0375 (0.0186–0.0785) | 0.0142 (0.0078–0.0430) | 0.000605 | 0.000970 | PSCI higher |
| ≥0.5% | ≥30% | 15 | 0.0375 (0.0186–0.0785) | 0.0142 (0.0078–0.0430) | 0.000605 | 0.000970 | PSCI higher |
| ≥0.5% | ≥40% | 15 | 0.0375 (0.0186–0.0785) | 0.0142 (0.0078–0.0430) | 0.000605 | 0.000970 | PSCI higher |
| ≥1.0% | ≥20% | 12 | 0.0368 (0.0182–0.0783) | 0.0142 (0.0077–0.0426) | 0.000647 | 0.000970 | PSCI higher |
| ≥1.0% | ≥30% | 12 | 0.0368 (0.0182–0.0783) | 0.0142 (0.0077–0.0426) | 0.000647 | 0.000970 | PSCI higher |
| ≥1.0% | ≥40% | 12 | 0.0368 (0.0182–0.0783) | 0.0142 (0.0077–0.0426) | 0.000647 | 0.000970 | PSCI higher |
| ≥2.0% | ≥20% | 9 | 0.0332 (0.0151–0.0768) | 0.0138 (0.0069–0.0403) | 0.001559 | 0.001559 | PSCI higher |
| ≥2.0% | ≥30% | 9 | 0.0332 (0.0151–0.0768) | 0.0138 (0.0069–0.0403) | 0.001559 | 0.001559 | PSCI higher |
| ≥2.0% | ≥40% | 9 | 0.0332 (0.0151–0.0768) | 0.0138 (0.0069–0.0403) | 0.001559 | 0.001559 | PSCI higher |

OES was calculated as the summed gut relative abundance of oral-associated genera selected under each threshold combination. The primary threshold used in the main analysis was oral mean abundance ≥1.0% and oral prevalence ≥30%. *P* values were calculated using the Wilcoxon rank-sum test, and q values were obtained using Benjamini–Hochberg correction across the nine threshold combinations. OES values are presented as relative abundance

***Supplementary Table 8. Performance of oral microbiota-based machine-learning models in the test set***

|  | **AUC (95% CI)** | **AP (95% CI)** | **Sensitivity** | **Specificity** | **Accuracy** | **Balanced accuracy** | **PPV** | **NPV** | **F1** | **Brier score** | **LogLoss** |
| --- | --- | --- | --- | --- | --- | --- | --- | --- | --- | --- | --- |
| RF | 0.866 (0.716–0.976) | 0.869 (0.755–0.973) | 0.895 | 0.750 | 0.821 | 0.822 | 0.773 | 0.882 | 0.829 | 0.138 | 0.453 |
| SVM | 0.811 (0.653–0.945) | 0.730 (0.604–0.948) | 0.632 | 0.800 | 0.718 | 0.716 | 0.750 | 0.696 | 0.686 | 0.179 | 0.545 |
| XGBoost | 0.793 (0.629–0.932) | 0.796 (0.657–0.946) | 0.789 | 0.800 | 0.795 | 0.795 | 0.789 | 0.800 | 0.789 | 0.245 | 0.684 |
| LR | 0.747 (0.574–0.897) | 0.747 (0.599–0.924) | 0.789 | 0.700 | 0.744 | 0.745 | 0.714 | 0.778 | 0.750 | 0.247 | 0.688 |
| NNET | 0.747 (0.582–0.895) | 0.747 (0.610–0.906) | 0.684 | 0.800 | 0.744 | 0.742 | 0.765 | 0.727 | 0.722 | 0.201 | 0.589 |
| LightGBM | 0.589 (0.397–0.766) | 0.641 (0.491–0.805) | 0.474 | 0.450 | 0.462 | 0.462 | 0.450 | 0.474 | 0.462 | 0.249 | 0.691 |

AUC, area under the receiver operating characteristic curve; AP, average precision; PPV, positive predictive value; NPV, negative predictive value; RF, random forest; SVM, support vector machine; LR, logistic regression; NNET, neural network; LightGBM, light gradient boosting machine. AUC and AP 95% confidence intervals were estimated using stratified bootstrap resampling with 2,000 resamples. Sensitivity, specificity, accuracy, balanced accuracy, PPV, NPV, and F1 score were calculated in the held-out test set using thresholds derived from training repeated cross-validation out-of-fold predictions based on the Youden index. Brier score and LogLoss were calculated using predicted probabilities in the held-out test set. Lower Brier score and LogLoss values indicate better probabilistic calibration.

***Supplementary Table 9. Performance of gut microbiota-based machine-learning models in the test set***

|  | **AUC (95% CI)** | **AP (95% CI)** | **Sensitivity** | **Specificity** | **Accuracy** | **Balanced accuracy** | **PPV** | **NPV** | **F1** | **Brier score** | **LogLoss** |
| --- | --- | --- | --- | --- | --- | --- | --- | --- | --- | --- | --- |
| LR | 0.871 (0.737–0.974) | 0.867 (0.731–0.974) | 0.684 | 0.850 | 0.769 | 0.767 | 0.812 | 0.739 | 0.743 | 0.247 | 0.687 |
| NNET | 0.866 (0.732–0.968) | 0.865 (0.731–0.970) | 0.842 | 0.800 | 0.821 | 0.821 | 0.800 | 0.842 | 0.821 | 0.164 | 0.508 |
| RF | 0.832 (0.679–0.953) | 0.749 (0.585–0.955) | 0.789 | 0.850 | 0.821 | 0.820 | 0.833 | 0.810 | 0.811 | 0.170 | 0.524 |
| SVM | 0.824 (0.676–0.947) | 0.727 (0.568–0.952) | 0.737 | 0.800 | 0.769 | 0.768 | 0.778 | 0.762 | 0.757 | 0.184 | 0.552 |
| XGBoost | 0.764 (0.596–0.913) | 0.728 (0.559–0.907) | 0.579 | 0.850 | 0.718 | 0.714 | 0.786 | 0.680 | 0.667 | 0.194 | 0.606 |
| LightGBM | 0.705 (0.542–0.853) | 0.725 (0.559–0.861) | 0.684 | 0.700 | 0.692 | 0.692 | 0.684 | 0.700 | 0.684 | 0.236 | 0.665 |

AUC, area under the receiver operating characteristic curve; AP, average precision; PPV, positive predictive value; NPV, negative predictive value; RF, random forest; SVM, support vector machine; LR, logistic regression; NNET, neural network; LightGBM, light gradient boosting machine. AUC and AP 95% confidence intervals were estimated using stratified bootstrap resampling with 2,000 resamples. Sensitivity, specificity, accuracy, balanced accuracy, PPV, NPV, and F1 score were calculated in the held-out test set using thresholds derived from training repeated cross-validation out-of-fold predictions based on the Youden index. Brier score and LogLoss were calculated using predicted probabilities in the held-out test set. Lower Brier score and LogLoss values indicate better probabilistic calibration.

***Supplementary Table 10. Performance of combined oral–gut microbiota-based machine-learning models in the test set***

|  | **AUC (95% CI)** | **AP (95% CI)** | **Sensitivity** | **Specificity** | **Accuracy** | **Balanced accuracy** | **PPV** | **NPV** | **F1** | **Brier score** | **LogLoss** |
| --- | --- | --- | --- | --- | --- | --- | --- | --- | --- | --- | --- |
| XGBoost | 0.945 (0.863–0.995) | 0.943 (0.867–0.995) | 0.789 | 0.950 | 0.872 | 0.870 | 0.938 | 0.826 | 0.857 | 0.105 | 0.348 |
| LR | 0.934 (0.853–0.989) | 0.935 (0.857–0.990) | 0.895 | 0.800 | 0.846 | 0.847 | 0.810 | 0.889 | 0.850 | 0.139 | 0.444 |
| NNET | 0.926 (0.837–0.987) | 0.924 (0.845–0.985) | 0.789 | 0.800 | 0.795 | 0.795 | 0.789 | 0.800 | 0.789 | 0.153 | 0.522 |
| SVM | 0.916 (0.816–0.984) | 0.926 (0.850–0.984) | 0.737 | 0.850 | 0.795 | 0.793 | 0.824 | 0.773 | 0.778 | 0.140 | 0.410 |
| RF | 0.908 (0.803–0.982) | 0.897 (0.799–0.979) | 0.842 | 0.800 | 0.821 | 0.821 | 0.800 | 0.842 | 0.821 | 0.129 | 0.409 |
| LightGBM | 0.889 (0.766–0.979) | 0.902 (0.808–0.983) | 0.895 | 0.800 | 0.846 | 0.847 | 0.810 | 0.889 | 0.850 | 0.166 | 0.514 |

AUC, area under the receiver operating characteristic curve; AP, average precision; PPV, positive predictive value; NPV, negative predictive value; RF, random forest; SVM, support vector machine; LR, logistic regression; NNET, neural network; LightGBM, light gradient boosting machine. AUC and AP 95% confidence intervals were estimated using stratified bootstrap resampling with 2,000 resamples. Sensitivity, specificity, accuracy, balanced accuracy, PPV, NPV, and F1 score were calculated in the held-out test set using thresholds derived from training repeated cross-validation out-of-fold predictions based on the Youden index. Brier score and LogLoss were calculated using predicted probabilities in the held-out test set. Lower Brier score and LogLoss values indicate better probabilistic calibration.
